# Gradual Not Sudden Change: Multiple Sites of Functional Transition Across the Microvascular Bed

**DOI:** 10.3389/fnagi.2021.779823

**Published:** 2022-02-14

**Authors:** Kira Shaw, Katie Boyd, Silvia Anderle, Matthew Hammond-Haley, Davina Amin, Orla Bonnar, Catherine N. Hall

**Affiliations:** ^1^Sussex Neuroscience, School of Psychology, University of Sussex, Falmer, United Kingdom; ^2^Brighton and Sussex Medical School, Brighton, United Kingdom; ^3^Department of Neuroscience, Physiology and Pharmacology, University College London, London, United Kingdom; ^4^MassGeneral Institute for Neurodegenerative Disease, Massachusetts General Hospital and Harvard Medical School, Charlestown Navy Yard, MA, United States

**Keywords:** neurovascular, pericyte, brain, mural cell, arteriole, capillary, endothelial cell

## Abstract

In understanding the role of the neurovascular unit as both a biomarker and target for disease interventions, it is vital to appreciate how the function of different components of this unit change along the vascular tree. The cells of the neurovascular unit together perform an array of vital functions, protecting the brain from circulating toxins and infection, while providing nutrients and clearing away waste products. To do so, the brain’s microvasculature dilates to direct energy substrates to active neurons, regulates access to circulating immune cells, and promotes angiogenesis in response to decreased blood supply, as well as pulsating to help clear waste products and maintain the oxygen supply. Different parts of the cerebrovascular tree contribute differently to various aspects of these functions, and previously, it has been assumed that there are discrete types of vessel along the vascular network that mediate different functions. Another option, however, is that the multiple transitions in function that occur across the vascular network do so at many locations, such that vascular function changes gradually, rather than in sharp steps between clearly distinct vessel types. Here, by reference to new data as well as by reviewing historical and recent literature, we argue that this latter scenario is likely the case and that vascular function gradually changes across the network without clear transition points between arteriole, precapillary arteriole and capillary. This is because classically localized functions are in fact performed by wide swathes of the vasculature, and different functional markers start and stop being expressed at different points along the vascular tree. Furthermore, vascular branch points show alterations in their mural cell morphology that suggest functional specializations irrespective of their position within the network. Together this work emphasizes the need for studies to consider where transitions of different functions occur, and the importance of defining these locations, in order to better understand the vascular network and how to target it to treat disease.

## Introduction

The human brain has an extensive vascular network essential for supplying the brain’s rich energy demands. Oxygen, glucose and other nutrients and signaling molecules are sent to the brain *via* finely regulated cerebral blood flow which is delivered through arteries, arterioles and capillaries. Deoxygenated blood and waste products are then removed *via* capillaries, venules and veins. In the neocortex, large pial vessels run along the surface then dive and penetrate the brain, before branching into smaller vessels to form the dense capillary network (Duvernoy et al., 1981). Pial and parenchymal arterioles and capillaries respond to neuronal nutrient and oxygen demand by dilating and contracting (Iadecola, 2017) to alter blood flow locally, a process called neurovascular coupling (NVC). NVC is controlled by the neurovascular unit (NVU), formed by vascular cells (mural cells - smooth muscle cells and pericytes, and endothelial cells), astrocytes and neurons. In addition to modulating contractile tone and blood flow, the NVU is also fundamental in regulating blood brain barrier (BBB) permeability and nutrient delivery (Hawkins and Davis, 2005; Iadecola, 2017; Sweeney et al., 2018), as well as helping to coordinate the brain’s immune response by restricting leukocyte invasion into the tissue.

Different parts of the vascular tree contribute differentially to these various functions, but in many cases, it remains unclear how function changes along the vessel bed. This is partly because of variability in nomenclature and definitions across studies, and partly because of an understandable, but ultimately misleading, tendency to oversimplify the manner in which these functional transitions occur. It is important to better understand these functional transitions, however, in order to identify which cells and vessels need to be targeted to manipulate these various processes therapeutically in conditions such as Alzheimer’s disease or stroke.

Here we use existing literature and novel data to consider which components of the vasculature mediate different functions, to better understand functional transitions across the vascular network. We suggest that evidence indicates multiple transition points at different positions in the vascular network, and therefore to gradual, heterogeneous changes in vascular function, rather than sudden transition points between distinct vessel types.

### Transitions in Anatomy Across the Cerebral Microvascular Bed

Across the vascular bed, from arteriole to capillary to venule, the diameter of vessels first decreases then increases, and the morphology of their mural cells (smooth muscle cells or pericytes) abluminal to the endothelial tube changes. Penetrating arteries and arterioles are typically between 15 and 40 μm in diameter and are composed of an inner layer of endothelial cells with abundant caveolae. They contain an internal elastic lamina and therefore express elastin in the vessel wall, which can be detected from its binding to the dye Alexa 633 hydrazide (Shen et al., 2012). At the surface, they often have 2-3 outer layers of closely packed ring-shaped smooth muscle cells (SMCs), which are electrically coupled *via* gap junctions (Chow et al., 2020; Garcia and Longden, 2020) and strongly express smooth muscle alpha actin (αSMA). At the pial surface, the blood vessel is separated from the pial membrane by Virchow-Robin space, which narrows after the arteriole enters the brain through a “pial funnel” (Gao et al., 2015). Downstream from this region, SMCs form only one layer before transitioning to a pericyte morphology. Grant et al. (2019) report the first cells with “bump on a log” morphology as always occurring beyond the first branch from the arteriole, and most reports consider the arterioles to be covered with annular SMCs (e.g., Hamilton et al., 2010; Grant et al., 2019; Kisler et al., 2017). However, even near the pial surface, mural cells’ morphology changes from a simple annulus to gaining a distinct soma and processes. While these cells are often still termed smooth muscle cells, historically this transitional form would be called a pericyte (Zimmermann, 1923; Attwell et al., 2016).

At the first branch point from penetrating arterioles are precapillary sphincters: contractile mural cells encircling a narrow section of vessel between the penetrating arteriole and first order capillary (Chambers and Zweifach, 1946; Grubb et al., 2020). The first branches off the penetrating arteriole are often termed precapillary arterioles or first order capillaries (where penetrating arterioles are branch order 0). These vessels tend to have a diameter of between 5-15 μm, and are enwrapped with mural cells that express αSMA and have a protruding, distinct soma, and processes that cover the vessel to a large degree, recently termed “ensheathing pericytes” (Hartmann et al., 2015; Grant et al., 2019; but see Hill et al., 2015; Attwell et al., 2016). These first branches are often termed the precapillary arteriole or transitional zone, though these terms sometimes are used to mean only the first vessel branching off the penetrating arteriole (e.g., Thakore et al., 2021), and other times include the 2nd and 3rd branches with larger pericyte coverage and αSMA actin expression (Gonzales et al., 2020).

Downstream of these vessels, after 1-3 branches from the diving arteriole (Grant et al., 2019) pericytes become less dense and their processes cover less of the vessel as branch order increases and vessel diameter decreases (range 3-9 μm). These capillary pericytes have been reported to have low (Bandopadhyay et al., 2001) or absent (Nehls and Drenckhahn, 1991; Grant et al., 2019) expression of αSMA and have been termed mesh and thin strand pericytes (Hartmann et al., 2015). Latterly these have been combined into a single category (capillary pericytes), due to the difficulty in distinguishing a transition point between these two morphologies (Grant et al., 2019). These capillaries with low or absent αSMA expression and low pericyte coverage are often termed mid-capillaries.

In post-capillary venules, pericytes have a stellate morphology, becoming less extended with thicker, more radial processes (Joyce et al., 1985; Hartmann et al., 2015). These post-capillary venules branch to form large venules (> 50 μm), which have weak αSMA labeling, indicating limited contractile potential (Hill et al., 2015). On larger venules, SMCs are circumferential but are less compact and show a less complete coverage of the vasculature and a leaf-like instead of banded appearance compared to arteriolar SMCs (Hill et al., 2015).

Therefore, the anatomy of the vessels and mural cells of the cerebral vasculature, as commonly reported, is used to define the following distinct vascular segments: pial arterioles, penetrating or diving arterioles, precapillary sphincters, pre-capillaries, also termed transitional or low branch order capillaries, mid-capillaries, post-capillaries and venules. These have been recently condensed into 4 categories, arterioles, a transitional zone, capillaries and venules, with distinct mural cells on each (Hartmann et al., 2021a). In some ways this categorization is a helpful way to discuss features of different parts of the vascular bed, as the recent Hartmann et al. review does in a clear and informative manner. Our argument, however, is that it is important to recognize that these categories are overly simplistic because they are superimposed on more gradually changing anatomy and function across the vascular bed. For example, existing literature shows that mural cell morphologies transition gradually not abruptly along the network. This concept was first discussed by Zimmermann (1923) and is evident from more recent work which shows that there is a continuum of cell lengths seen from SMCs through ensheathing to mid-capillary pericytes (Grant et al., 2019). Furthermore, the shift in vessel coverage of pericyte processes argued to mark a boundary between ensheathing and mesh pericytes has a different distribution across branch orders than that of αSMA expression (Grant et al., 2019), i.e., αSMA transitions occur at largely different locations than do transitions in pericyte coverage of the vessel.

### Neurovascular Coupling Differences Across the Cerebral Microvascular Bed

This heterogeneity of anatomy across the vascular tree presumably reflects the varying functional roles performed by different vessels. Anatomical transitions have been best mapped onto functional changes in terms of dilatory capacity and involvement in neurovascular coupling (i.e., dilation in response to increased neuronal activity). Disease affects different parts of the vasculature in specific ways, making it important to understand how and where such functional transitions occur to be able to intervene successfully to target pathological mechanisms.

Classically, neuronal activity causes local pial and penetrating arterioles to dilate, causing an increase in blood flow to active brain regions (Attwell et al., 2010). However, different components of the vasculature are now known to play distinct roles in generating this response. Mid-capillary regions, being closer on average than any other vessel segment to most neurons, likely first detect neuronal activity, either *via* the accompanying increase in extracellular potassium concentration (Longden et al., 2017) and/or *via* production of vasoactive signaling molecules such as prostaglandin E2 (Hall et al., 2014; Mishra et al., 2016) or EETs (Mishra et al., 2016; Zhang et al., 2021). This signal then spreads upstream from the capillary bed, causing dilation of upstream low branching order capillaries and arterioles (Hall et al., 2014; Rungta et al., 2018), *via* spread of hyperpolarizing currents carried by inward rectifying potassium channels (Zhang et al., 2011; Longden and Nelson, 2015). Whether mid-capillaries constrict and dilate in response to modulation of neuronal activity remains somewhat controversial. Some studies report that they do not (Hill et al., 2015), but a growing body of evidence supports their contractility (Hall et al., 2014; Kisler et al., 2017; Hartmann et al., 2021b; Shaw et al., 2021), though this may be *via* a different mechanism than in arterioles and lower branching order capillaries (Hartmann et al., 2021b).

The size, frequency and timing of responses to neuronal activity are often reported to vary across vascular compartments though not always in a consistent manner. Arteriole response magnitudes vary along their length, with surface sections of penetrating arterioles dilating more than deeper sections, probably as a result of the mechanical restriction imposed by brain tissue (Gao et al., 2015), though deeper arteriole sections have also been found to dilate more rapidly than shallower sections (Tian et al., 2010). In alpha-chloralose anaesthetized somatosensory cortex, Hall and colleagues found response frequency decreased as branching order increased (i.e., capillaries responded less frequently than arterioles; Hall et al., 2014), though first and second branch order capillaries showed larger and faster dilations than arterioles, a finding subsequently supported by other studies (Zhang et al., 2021). In the olfactory bulb, the region around the branch off the arteriole also dilated first, with slower responses up and downstream (Rungta et al., 2021). However, the same group found much more variable timings in neocortex of ketamine-medetomidine anaesthetized or awake mice, with capillaries or arterioles each being faster on some occasions (Rungta et al., 2021), and in both these reports, mid-capillary dilations were very small (though these data conflate responders and non-responders, unlike some other studies, Hall et al., 2014; Shaw et al., 2021). Precapillary sphincters, where studied, have often shown larger dilations, as a proportion of their diameter, than adjacent arterioles and capillaries (Grubb et al., 2020; Zambach et al., 2021).

In short, there are functional differences between neurovascular coupling responses along the vascular network, with mid-capillaries responding less frequently than upstream vessels to increases in local neuronal activity, and the fastest responses often, but not exclusively, observed in the first and second order capillaries. The physiological mechanisms underlying this heterogeneity in contractile responses are not wholly clear. Pericytes expressing αSMA seem likely to mediate more reliable, often larger and faster changes in vascular tone (Hill et al., 2015; Hartmann et al., 2021b), but as αSMA is commonly expressed in vessels up to and including the 3rd branch from the penetrating arteriole (Grant et al., 2019), variations in αSMA expression alone cannot explain the observed pattern of responses.

Indeed, several vasoactive pathways and mechanisms modulating the diameter of different vascular segments have been found to vary across the vascular network. Vessels beyond the penetrating arteriole were found to dilate *via* ATP-mediated increases in astrocytic calcium and prostaglandin action on EP4 receptors, while diving arterioles dilated *via* NMDA receptor mediated NO production (Mishra et al., 2016). Another functional transition point at the same location was also recently observed, as capillary endothelial cells were found to express MFSD2A, inhibiting caveolae formation, while arteriolar endothelial cells showed much lower MFSD2A expression, instead having abundant caveolae that were necessary for intact neurovascular coupling (Andreone et al., 2017). Because MFSD2A-mediated reduction in caveolae formation reduces transcytosis to sustain BBB integrity (Ben-Zvi et al., 2014), this suggests a possible transition in both BBB function and neurovascular coupling mechanisms at the same arteriole-to-capillary point.

It is not just the mechanism of vasodilation that is sensitive to the position in the microvasculature: The integration of vascular signals across the vascular network is an area of increasing focus, with many recent papers shedding new insights on how vasoactive signals are propagated upstream of their detection in the mid-capillary bed. Potassium currents (Longden et al., 2017), nitric oxide (Kovacs-Oller et al., 2020), calcium and ATP signals (Thakore et al., 2021) have all recently been shown to be important in the transmission of vasodilatory signals and, by their nature, these signals span vascular segments and may be influenced by functional variations across these sections. While many studies have not explicitly investigated how different vascular segments affect signal transmission, Thakore et al. (2021) recently showed that ATP application or TRPA1 channel activation in the capillary bed produces slowly-propagating calcium signals through capillary endothelial cells that depend on ATP release from Panx1 channels, but are converted, by IK and SK channels in a transitional segment, into rapidly-propagating electrical signals that dilate upstream arterioles. A similarly propagating signal has been shown to be initiated in the capillary bed by PGE2 (Rosehart et al., 2021; this issue). In these papers, the transitional zone was defined as the first branch off the penetrating arteriole, and did not include lower branching order vessels that would be expected to express αSMA, and it is not clear whether these higher branching order vessels also show rapid IK or SK-mediated propagation of dilation.

Perhaps unsurprisingly given the varying vasoactive properties of different vascular segments, their function can be differentially affected by disease. In Alzheimer’s disease, capillaries may mediate functionally significant decreases in cerebral blood flow, as capillaries but not arterioles were found to be constricted due to endogenous Aβ-mediated endothelin signaling to pericytes (Nortley et al., 2019), and to be plugged by neutrophils (Cruz Hernández et al., 2019). Similar effects may be seen after stroke, with capillaries constricting in brain slices during ischemia (Hall et al., 2014) and showing stalled flow and constrictions after reperfusion *in vivo* (Yemisci et al., 2009), though the largest constrictions may be seen in αSMA-expressing vessels rather than mid-capillary regions (Hill et al., 2015). Conversely, in mice carrying the main genetic risk factor for Alzheimer’s disease, APOE4, capillary function was unaffected, but the large pial vessels were dysfunctional, with vasomotion and dilation frequencies reduced compared to APOE3-expressing controls (Bonnar et al., 2021). However, none of these studies define the precise location of these transitions in disease susceptibility.

To summarize, the contractile properties of the vascular tree, in terms of both capacity and timing of dilations, mechanisms mediating neurovascular signaling and integration, and the sensitivity of these processes to disease states all vary depending on the position in the vascular network. In some cases (e.g., response frequency) function seems to gradually change along the network, while in others (e.g., mechanism of neurovascular coupling), there are more abrupt transitions of function, and in many cases the location of functional transitions remains unknown.

### Oxygen Supply

The major function of the brain’s blood supply is to provide it with nutrients. These include the energy substrates, oxygen and glucose, as well as nucleosides and amino acids needed for mRNA and protein synthesis. Classically, the dense capillary bed has been thought of as the site of nutrient exchange, but actually measuring where nutrient delivery occurs is not straightforward.

Improved two-photon phosphorescence lifetime imaging of oxygen probes has recently provided insights into the concentration gradients of oxygen across the microvasculature in mouse cortex, and therefore where most oxygen delivery likely takes place. In anaesthetized cortex, arterioles were found to be responsible for 50% of the extracted oxygen (Sakadžić et al., 2014), with capillaries (here defined as vessels two branches downstream of the penetrating arteriole) appearing to provide a reserve for oxygen delivery capacity, taking on a larger role during hypercapnia and, as suggested by modeling, when oxygen consumption rates increased. Indeed, in more recent studies in awake mice (therefore with a larger basal oxygen consumption rate), less oxygen was found to be delivered by the arterioles (34%), with the majority of the remainder expected to be delivered by the capillary bed (Li et al., 2019).

The factors driving oxygen delivery by these different vessels include red blood cell linear density, speed, local oxygen concentration gradients, and intravascular resistance to oxygen diffusion. These differ between cortical layers (Lyons et al., 2016; Schmid et al., 2017; Li et al., 2019) and are differently variable within these layers. For example, red blood cell (RBC) flow was lowest and more homogenous between capillaries and, correspondingly, oxygen extraction was largest in deep cortical layers, with faster flow and less oxygen extraction in superficial capillaries (Schmid et al., 2017; Li et al., 2019). This was in part because blood pressure dropped more in the diving arteriole when feeding deeper layers (Schmid et al., 2017). Furthermore, from arterial to venous capillaries, oxygenation becomes increasingly variable as an increasingly clear relationship between RBC flow and pO2 emerges [more O2 having been extracted from capillaries with slowly moving RBCs (Li et al., 2019)]. Thus, position in the vascular network in terms of both tissue depth and branching order affects how blood delivers oxygen to the tissue.

### Nutrient Supply and the BBB

Unlike oxygen, which can diffuse through cell membranes, other nutrients are supplied to the brain *via* specialist transporters which allow their passage across the BBB. The conventional view is that arterioles and arteries control the blood supply to the tissue but do not participate in the exchange of nutrients in the brain, which is instead mediated by capillaries and post-capillary venules (Yuan and Rigor, 2010). The BBB strictly controls the influx and efflux of molecules across its endothelial layer largely by active transport. In addition to being vital for neuronal function, these proteins can be targeted for drug development to allow drugs to access the brain.

Little work has studied where in the vascular network these transporters are most active, though a recent RNA Seq study showed them to be predominantly expressed in capillary and venule mural cells, in contrast to transcription factors which were overrepresented at the arterial end of the vasculature (Vanlandewijck et al., 2018). A key question to be addressed in future, is whether this myriad of different transporters and transcription factors transition in similar or different locations across the vascular network.

### The BBB and Immune Regulation

The BBB is also a key site of regulation of immune access to the brain, which again varies across the vascular tree. Unlike arteriolar and capillary endothelial cells, post-capillary venular endothelial cells are connected by adherens rather than tight junctions, meaning they are more leaky than the capillaries which feed them (Rous and Smith, 1931; Pober and Sessa, 2014). The basement membrane that surrounds them also has a different structure, being lamellar instead of homogenous (Braverman, 1989). Leukocyte infiltration into the surrounding tissue primarily happens in this region, leukocytes migrating through gaps between pericytes and thus being regulated by pericyte morphology (Proebstl et al., 2012). Pericyte contractility may feed into this process, as pericyte relaxation (not contraction) widens the gaps between cells, facilitating leukocyte infiltration (Wang et al., 2012). Once in the parenchyma, leukocytes migrate along NG2-expressing pericytes, which release factors to support their migration (Stark et al., 2013). Thus, an important transition in vascular immune function appears to occur between NG2-positive pericytes on capillaries and the NG2-negative pericytes on veins, though whether this happens to the same extent in brain as in peripheral tissues remains unknown. In addition to regulation of immune cell entry, the vasculature can also transport cytokines into the brain, as well as respond to circulating factors with parenchymal production of cytokines and chemokines (Banks et al., 2009; Rustenhoven et al., 2017), but how this varies across the vascular network is also unknown.

More studies into the site of immune regulation in the brain would be valuable, however, as, perhaps unsurprisingly, these pathways of immune regulation by pericytes and endothelial cells are important for understanding and treating disease. Pericyte damage and increased permeability of the brain vasculature to plasma proteins and immune cells are hallmarks of Alzheimer’s disease, multiple sclerosis, and stroke (Daneman, 2012), where infiltrating immune cells have also been shown to induce vascular dysfunction, demyelination, axonal damage, and neurodegeneration (Hochmeister et al., 2006; Ryu et al., 2015). Understanding where in the network structural and functional contributions of the vasculature to the regulation of neural inflammation occur may thus enable us to understand which cells should be best targeted therapeutically.

### Vasomotion

As well as nutrients being delivered across the BBB, the vasculature also plays an important role in the removal of potentially neurotoxic substances from the surrounding brain tissue. Vasomotion, a phenomenon first reported in bat wing veins (Jones, 1853), is a spontaneous low frequency oscillation in blood vessel tone (typically centered near 0.1 Hz; Mayhew et al., 1996); and independent of heartbeat or respiration), which is present in vessels (particularly arteries) throughout the body (Aalkjær et al., 2011). This rhythmic pulsing of vessels is thought to contribute substantially to paravascular clearance (Aldea et al., 2019; van Veluw et al., 2020) and tissue oxygenation (Tsai and Intaglietta, 1989; Aalkjær et al., 2011; Thorn et al., 2011; Mateo et al., 2017). In arterioles, oscillations are generated within the vascular wall as a result of local SMC dilations and contractions, possibly *via* phospholipase C and phospholipase A_2_ mediated cyclical release of calcium from IP3-sensitive stores (Haddock et al., 2002). Capillaries have not been reported to show these low frequency oscillations in diameter, though RBC flow does show similar fluctuations which may be due to upstream diameter fluctuations (Colantuoni et al., 1994; Biswal and Hudetz, 1996). However, a functional transition in vasomotion of vessel diameters has not yet been pinpointed.

Given the importance of vasomotion as a driving force for paravascular tissue clearance, it is not surprisingly affected by vascular-degenerating diseases. In a mouse model of Alzheimer’s disease, arterioles surrounded with Aβ showed impaired vasomotion when driven by neuronal activity, and showed reduced dextran clearance from the parenchyma (van Veluw et al., 2020). Mice carrying the main genetic risk factor for Alzheimer’s disease, APOE4, also showed a reduction in pial arteriole vasomotion compared to APOE3 controls (Bonnar et al., 2021). Thus, vasomotion is important for regulating supply and clearance of substances to the brain, and is affected by disease. However, we do not yet know where in the vascular tree the transition point falls in which the vessels cease to show vasomotion.

### Summary

Vascular anatomy, contractility, nutrient delivery, immune regulation and clearance all differ across the vascular bed. To a large extent, functions of the vascular network have been assumed to transition broadly with branching anatomy, from vasomotion pial and diving arterioles that show reliable, large neurovascular coupling responses, through fast responding pre-capillaries/low branching order capillaries, to less reliably dilating mid-capillaries. These mid-capillaries are thought to have a stronger role than upstream vessels in BBB regulation and nutrient transport, while further downstream, post-capillary venules regulate immune cell infiltration. While this framework no doubt captures the broad changes that occur over the vascular network, it is by no means clear that the vasculature can be usefully divided into such discrete divisions. Indeed some of the research discussed above points to the existence of additional functional transitions within vascular segments, including the decreasing size of dilations in superficial and deeper diving arterioles (Gao et al., 2015), and pericyte coverage of vessels continuing to decrease into the capillary bed (Grant et al., 2019). However, most focus on localizing functional changes across the vasculature has fallen on contractile function or mural cell anatomy, so it is unclear whether other functions transition at the same, or different, locations. If different functions show the same transition points as contractile function, we could reasonably consider the vascular segments separated by these transition points as different vessel types. If, however, different vascular functions transition at different locations, it becomes somewhat meaningless to use these shifts in contractile function to define vascular segments.

To test the principle of whether there are clear locations within the vascular network at which transitions occur between multiple functions of the vasculature, we used immunohistochemistry to label different functional markers in brain slices expressing DsRed under the control of the NG2 promoter, as well as *in vivo* two-photon imaging of vascular responses to visual stimuli in awake mice to study the contractile characteristics of the vascular bed at the different functional transition points. There was no single point in the vasculature where the function transitioned from “arteriole-like” to “capillary-like.” Classic pericyte markers, NG2 and PDGFRβ were in fact expressed throughout the vascular network, while transitions in markers for contractility, arteriolar compliance, neurovascular coupling and angiogenic potential occurred at different points in the vascular bed. We observed no sudden transitions in mural cell morphology across the vascular network, or at termination points of functional markers, though branch points showed distinct changes in mural cell morphology throughout the vasculature studied (arteriole-capillary). Finally, we also observed transitions of neurovascular and vasomotion function down the depth of arterioles, highlighting that arterioles become more capillary-like further from the pial surface.

## Materials and Methods

### Animals

All experimental procedures were performed in accordance with the 1986 Animal (Scientific Procedures) Act and approved by the United Kingdom Home Office and the University of Sussex or University College London animal welfare ethical review boards. Experiments were performed on mice aged 1-8 months of both sexes (3-8 months for *in vivo* experiments). Mice were all on a C57BL/6J background which were either wild-type, expressed DsRed under the control of the NG2 promoter (Zhu et al., 2008), or for data collected from the 186 vessels recorded for the *in vivo* experiments expressed GCaMP6f under the control of the Thy1 promoter (Dana et al., 2014) or were SST-Cre crossed with floxed GCaMP6f. Food and water were available *ad libitum*, and mice were housed at 22°C in a 12 h light/dark cycle (which was reversed for the mice used in *in vivo* experiments).

### *Ex vivo* Imaging

Each immunohistochemistry experiment used tissue from a minimum of 3 animals, on at least 3 different experimental days. Methods for slicing, fixing and labelling were as described previously (Mishra et al., 2014; Boyd et al., 2021), as follows:

#### Slicing

NG2 DsRed mice were sacrificed by Schedule 1 approved methods (cervical dislocation followed by decapitation). The brain was then removed from the cranium and anterior and posterior coronal sections were removed, producing a block containing the central portion of the cerebral hemispheres. This block was then mounted onto a chilled slicing block using cyanoacrylate glue, with the inferior surface of the brain facing an agarose block. Using a vibratome, 200 μm coronal brain slices were prepared in ice-cold slicing solution containing (mM): NaCl (124), NaHCO3 (26), glucose (10), KCl (2.5), MgCl2 (2), CaCl2 (2), NaH2PO4 (1), kynurenic acid (1), bubbled with 95% oxygen and 5% CO2. Slices were incubated in a slice storage container containing slicing solution bubbled with 95% oxygen and 5% CO2 at room temperature to recover for > 30 min before fixation. Some slices were incubated in oxygenated slicing solution containing the fluorescent dye AlexaFluor 633 hydrazide (20 μM) to label elastin (Shen et al., 2012).

#### Fixation, Blocking and Permeabilization

Slices were fixed in 4% paraformaldehyde solution for 30 min in a 24-well plate on a rotary shaker. Slices were then washed (3 × 10 min) in 0.1 M phosphate buffered saline (PBS) on a rotary shaker at room temperature. In preparation for antibody labeling the slices were incubated for 3 h in 0.2% Triton X-100, 10% normal goat serum and 0.2M glycine in 0.1M PBS (blocking and permeabilizing solution) at room temperature to block generalized secondary antibody staining and to permeabilize the tissue.

#### Immunohistochemical Labeling

The following primary antibodies were used, all at 1:200 dilution in 0.1M PBS: rabbit anti-αSMA (Abcam), chicken anti-nestin (Abcam), rabbit anti-PDGFRβ (Santa Cruz Biotechnologies), rabbit anti-GLUT-1 (Abcam), and chicken anti-GFAP (Abcam). Slices were incubated in primary antibody in PBS overnight (12-16 h) on a rotary shaker at room temperature, washed (3 × 10 min) in 0.1 M PBS, followed by a 6-8 h fluorescently labeled appropriate secondary antibody incubation in 0.1M PBS at room temperature (AlexaFluor 647 goat anti-rabbit IgG or AlexaFluor 488 donkey anti-rabbit IgG - both Life Technologies; Goat anti-chicken CF488 IgY - Sigma). A no-primary control experiment was used for each antibody to check for non-specific binding of secondary antibodies. After labeling, slices were mounted onto microscope slides with Vectashield hard-set mounting medium including DAPI (Vector Laboratories) and covered with a glass cover slip. Cover slip borders were then sealed with nail varnish.

#### Confocal Imaging

SMCs and pericytes on large and small blood vessels, respectively, were readily identifiable through DsRed expression under the control of the NG2 promoter. Fluorescent labeling of the proteins described previously allowed visualization of the expression of proteins involved in the various functions of the cerebral vasculature. Fixed slices were imaged using a Leica SP8 or Zeiss LSM780 confocal laser-scanning microscope. Imaging with multiple wavelengths was performed as sequential scans at each wavelength, to minimize “bleed-through” of fluorescence between channels. Z stacks of penetrating arterioles and their downstream capillary bed were obtained using a 20x air objective. These stacks were then projected as ‘maximum-intensity projections’ and stitched together using the MosaicJ plugin of ImageJ software (NIH, Bethesda, MD, United States). High-power Z-stacks of regions of interest, including the transition point of the various markers, were acquired using a 63x oil immersion objective.

#### Image Analysis

Image analysis was conducted using FIJI/ImageJ. Intersoma distances and diameters were calculated manually using the measurement tool in ImageJ. Measurements were made in one z-plane by drawing a line from the mid-point of the pericyte soma nearest the transition in labeling, to the mid-point of the closest proximal vascular mural cell on the same vessel. Somata were identified by DsRed expression and confirmed by DAPI stained cell nuclei. Diameters were measured as the distance between the innermost DsRed labeling on either side of the vessel lumen (i.e., the diameters include endothelial cell thickness). Branch points of the vessels were identified from the penetrating arteriole, termed branch order 0, incrementing by 1 at each subsequent branch point.

### *In vivo* Imaging

#### Surgery and Two-Photon Imaging

Cranial window surgery to insert an optical window over V1 suitable for chronic, awake two-photon imaging was performed on 11 mice (6 female) under isoflurane anesthesia (see Shaw et al., 2021 for full methods). Following a one-week minimum post-surgery recovery period, mice were habituated to head fixation over multiple sessions. The experimental set-up consisted of a cylinder fitted with a Kuebler rotary encoder placed under a two-photon microscope with red and green filters (Scientifica), and in front of two computer screens for visual stimulus presentation. The objective used for two-photon imaging was a water-based 16x aperture (LWD, Nikon) with tissue excited at 940 nm (Chameleon Vision II Ti:Sapphire laser, Coherent). The visual stimulus was a drifting grating (PsychoPy) that varied either by contrast (25, 63, or 100%, all at 315° orientation), size (20° small circular stimulus or 220° full screen stimulus, both at 100% contrast), or spatial frequency (0.04 or 0.2 cycles per degree). As our data selected only the trials where the mouse was not running, chi-square tests were conducted to ensure all vessel segments were subject to the same distribution of stimulus conditions across trials (contrast *p* = 0.94; size *p* = 0.99; spatial frequency *p* = 0.99). Imaging sessions recorded fluorescent blood vessels and calcium activity (in excitatory or somatostatin cells), however, for the purposes of this study only data recorded from vessels was analyzed. To visualize blood vessels (penetrating arterioles and their downstream capillaries) during darkness or visual stimulation whilst the animal was running or resting, mice were injected with 2.5% (w/v) Texas Red Dextran dissolved in saline (70 kDa *via* tail vein or 3 kDa subcutaneously, Fisher Scientific). For the stimulus-dependent vessel dilation data, we looked only at the dilations occurring during rest trials (i.e., to remove locomotion confounds). Imaging sessions were recorded in SciScan software (SciScan v1.2.1, Scientifica), where the imaged vessels ranged between 0 and 729.6 μm in depth (mean: 182.55 μm, SD: 132.65 μm), had an average pixel size of 0.1958 μm (range: 0.1484 - 0.4431 μm, SD: 0.08 μm) and were acquired at speeds of 7.6 Hz.

#### Vessel Classification

Vessel diameter, vessel depth and branch order were measured for all vessel classifications (see Table 1). Because we wanted to look at responses down the penetrating arteriole, in these experiments we separately classified the penetrating arteriole and capillary branching orders. This was therefore different to that used for immunohistochemical analysis, where the penetrating arteriole was always 0, and branch orders always referred to the position in the capillary bed. Here, for penetrating arterioles, branch order started at 0 at the pial surface, and increased by 1 down the length of the diving vessel after a branch offshoot was encountered. For capillaries, the first offshoot protruding off the penetrating arteriole was always given a branch order of 1 and vessel branch order increased by 1 for each bifurcation encountered (see Supplementary Figure 1).

**TABLE 1:** Vessel characteristics/responses separated by vascular segment.

Vessel Label	Branch Order	Sample size	Num Trials	Diameter (μm)	Depth (μm)	Pixel size (μm)	Response rate (%)	Peak dilation (%)	Peak dilatio*n* (μm)	Power at 0.1Hz
Penetrating Arteriole	0	21 vessels (20 for Welch’s)	5-19 (mean: 12.86)	10.14 ± 4.66	74.40 ± 48.31	0.2438 ± 0.096	35.51 ± 25.36	All: 5.59 ± 5.20 R: 12.50 ± 17.80	All: 0.47 ± 0.05 R: 0.66 ± 0.11	0.040 ± 0.05
Penetrating Arteriole	1	22 vessels	2-17 (mean: 11.23)	9.62 ± 4.91	133.39 ± 78.07	0.2351 ± 0.078	26.99 ± 26.95	All: 4.69 ± 3.29 R: 7.90 ± 4.56	All: 0.35 ± 0.03 R: 0.34 ± 0.06	0.025 ± 0.03
Penetrating Arteriole	2	12 vessels	3-17 (mean: 12.33)	8.60 ± 2.35	130.03 ± 90.50	0.2025 ± 0.053	19.66 ± 14.22	All: 3.14 ± 1.48 R: 5.25 ± 2.86	All: 0.43 ± 0.04 R: 0.40 ± 0.07	0.017 ± 0.03
Penetrating Arteriole	3 +	9 vessels (8 for Welch’s)	11-17 (mean: 13.78)	11.31 ± 4.65	234.14 ± 62.05	0.2528 ± 0.089	13.87 ± 11.35	All: 3.03 ± 1.90 R: 3.32 ± 3.42	All: 0.75 ± 0.1 R: 0.86 ± 0.15	0.017 ± 0.02
Capillary	1	50 vessels (49 for Welch’s)	2-17 (mean: 11.24)	6.72 ± 3.36	161.51 ± 139.57	0.1603 ± 0.026	18.89 ± 18.28	All: 5.47 ± 6.69 R: 9.29 + /8.99	All: 0.37 ± 0.03 R: 0.42 ± 0.07	0.025 ± 0.06
Capillary	2	29 vessels	3-19 (mean: 12.41)	5.84 ± 2.02	161.86 ± 139.19	0.1595 ± 0.022	17.10 ± 16.23	All: 5.22 ± 3.84 R: 7.90 ± 8.79	All: 0.34 ± 0.03 R: 0.36 ± 0.07	0.027 ± 0.05
Capillary	3	22 vessels	4-19 (mean: 12.14)	5.75 ± 1.81	182.96 ± 100.9	0.1541 ± 0.017	25.14 ± 27.40	All: 4.28 ± 2.67 R: 7.00 + /4.98	All: 0.28 ± 0.04 R: 0.24 ± 0.06	0.011 ± 0.02
Capillary	4 +	21 vessels	2-18 (mean: 11.43)	4.84 ± 1.62	219.14 ± 92.02	0.1564 ± 0.020	18.53 ± 22.92	All: 9.04 ± 11.79 R: 7.14 ± 4.56	All: 0.22 ± 0.03 R: 0.14 ± 0.03	0.009 ± 0.01

*Values are calculated per individual vessel (presented as mean ± STD between vessels). All: averaged across all trials; Raveraged across responsive trials only. Note that some sample sizes are lower for the Welch’s power spectrum comparisons (specified in brackets) as 3 vessels were removed as outliers (see methods for outlier details).*

#### Data Extraction

To improve image quality, vessel recordings were subject to several preprocessing steps prior to analysis. Using ImageJ: image type was set at 8 bit, images were despeckled, and light artifacts from the stimulus minimized using the ‘stack contrast adjustment’ plugin. To correct for motion artifacts (mainly arising from locomotion) vessel recordings were also image registered using Suite2P (Pachitariu et al., 2016). A custom MATLAB script was used to extract vessel diameter along the full width at half maximum (see Shaw et al., 2021). Diameter measurements were averaged along the entire vessel length, leaving an averaged (per frame) continuous diameter trace over time.

#### Data Analysis

*Stimulus-dependent responses:* Continuous vessel diameter traces were cut into 30 s trials around the stimulus presentations (stimulation occurred between 5-10 s). Data trials were then normalized to the 5 s pre-stimulus baseline period (ΔD/D = (D-Dmin)/(Dmax – Dmin)), and multiplied by 100 (to present data as a% increase from baseline). Only ‘rest’ trials were included, meaning there was no significant locomotion occurring in the 2 s prior to or during the stimulus. Locomotion events had a duration > 1/3 s and were distanced from other locomotion epochs by at least 1 s. The locomotion-free stimulus-centered vessel trials were categorized as responsive or non-responsive to stimulation, with responsive trials being those in which the maximum dilation during the stimulus event exceeded 0.5 × the standard deviation of the 5 s baseline period. The maximum dilation was calculated by finding the peak of the trace during the stimulus. The peak at the cessation of the stimulus was taken by measuring the value of the vessel dilation trace at 10 s. The onset time of dilation responses was calculated as the time to 10% of the maximum peak (during the stimulation period). Four noisy data trials (from 2222 total trials) were removed from the diameter traces [3 trials from vessel 77 (C5) and 1 trial from vessel 167 (C5)] because dilation shifts were very noisy (high frequency shifts reflective of motion artifacts/signal loss) and peaks exceeded 10x the average (mean dilation peak across all responsive vessels during visual stimulation: 8.43 vs 101.3%, 123.2, 214.3, and 92.4%).

#### Power Spectra Responses

Welch’s power spectral density estimates were computed across all continuous vessel diameter traces. These recordings could be during rest, visual stimulus and/or locomotion, and we included the entire continuous trace in the power analysis as (a) the stimulus presentation occurred for 5 s every 30 s, meaning the power spectrum peak corresponding to stimulus presentation would be at 0.03 Hz (outside our 0.05-0.15 Hz range of interest); and (b) there was no significant difference in the amount of time spent in locomotion between the different vessel categories (mean time spent in locomotion: 13.07%, standard deviation: 6.39%, *p* = 0.45). All continuous data was detrended by subtracting the baseline (the 8th percentile calculated over a 15 s time window; see Bonnar et al., 2021). The MATLAB function “pwelch” was used to conduct discrete Fourier transforms across 60 s time windows. Data plots display raw power spectra and power spectra corrected for 1/f (pink) noise (1/f corrected trace = Welch power spectrum trace * corresponding Welch frequency trace). All data underwent outlier removal based on the maximum value detected between 0-1 Hz (all traces containing peaks greater than 3 * the standard deviation of all the maximum values were removed). This resulted in the removal of 3/186 vessel diameter traces (from categories: PA BO0, PA BO3, and Cap BO1). For comparing power spectra, the power value at 0.1 Hz was extracted. The number of vessels which showed high power at 0.1 Hz was assessed by separating vessel diameter traces by those above and below a set threshold (standard deviation across all detected power values at 0.1 Hz * 0.5).

#### Statistics

Statistical analyses were conducted in RStudio. Data in graphs are presented as mean ± SEM, and individual dots on bar graphs represent data from individual blood vessels, whereas violin plots were used to represent individual stimulus-presentation trials. For comparing the distribution of vessel responsivity rates or stimulus condition, data was taken from individual stimulus-presentation trials, and tested using a 3D Cochran-Mantel-Haenszel test with Fisher’s *post hoc* comparison. For comparing the sizes of vessel dilations or the power in the vasomotion frequency range, where data was non-normal, data was averaged across vessels and a Kruskal-Wallis test with *post hoc* pairwise Wilcoxon rank sum tests were utilized. Branch order and/or vessel categorization (penetrating arteriole/capillary) were set as the independent variable, dilation peak (for all trials during stimulus period or at stimulus cessation), time to 10% of dilation peak, power (value at 0.1 Hz or AUC between 0.05-0.15 Hz) or locomotion frequency (% time spent in locomotion) as the dependent variable. For comparing the individual vessel diameters and depths, where data was normally distributed, one-way ANOVAs with Tukey’s *post hoc* comparisons were used. For *post hoc* tests, for clarity only significant (*p* < 0.05) or trend (*p* < 0.1) level comparisons are shown, but these tests were always carried out, so absence of a displayed *p* value indicates a non-significant (*p* > 0.1) result.

## Results

### “Pericyte Markers” Are Expressed Throughout the Microvascular Network

**FIGURE 1:**
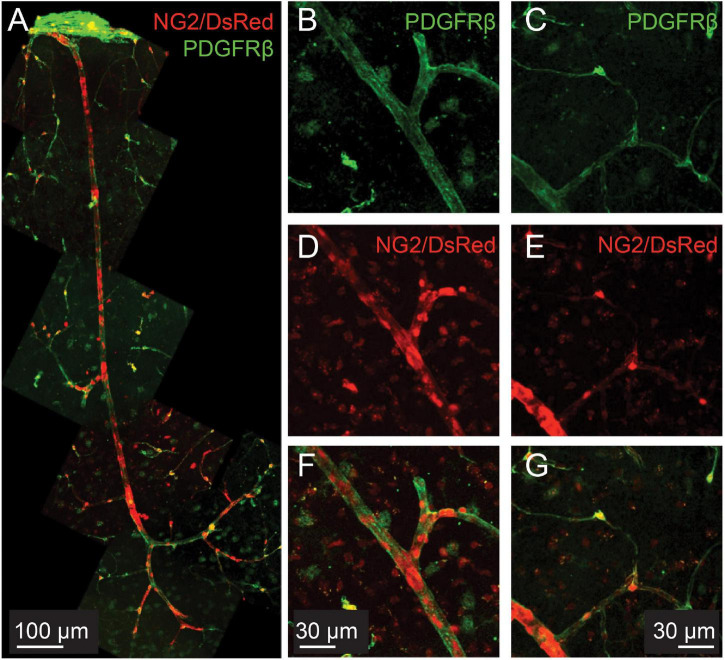
PDGFRβ is expressed in smooth muscle cells as well as pericytes in the cortical microvasculature. Green: immunohistochemical labeling for PDGFRβ. Red: DsRed expressed under the control of the NG2 control promoter. **(A)** Example vascular bed from pial surface (top) to layers V/VI of cortex (bottom). **(B–G)**: Two different example vessels at higher magnification, to show capillary pericyte labeling of both markers.

NG2, a chondroitin sulfate proteoglycan, is considered a pericyte marker and has been reported at low levels in smooth muscle cells (Kumar et al., 2017). However, NG2-controlled DsRed labeling is found throughout the microvascular tree, including in smooth muscle cells with a clear banded morphology (Figure 1). Immunohistochemical labeling for another pericyte marker, PDGFRβ (Winkler et al., 2010), also reveals protein expression throughout the microvascular tree, including in SMCs on pial vessels as well as penetrating arterioles and capillaries (Figure 1).

### Markers of Contractility and Elasticity Terminate at Different Positions of the Vascular Tree

As discussed above, a much studied transition across the vascular bed has been that between vascular mural cells expressing high levels of αSMA and those expressing low or no levels of αSMA, which broadly occurs between ensheathing and mesh pericytes, though the distribution of αSMA termination positions is different from that of the change in vessel coverage (Grant et al., 2019). As discussed above, this switch in αSMA expression may coincide with the faster responses observed in low branching order capillaries (Rungta et al., 2018), though contractility of pericytes may extend beyond this transition in αSMA expression, as mid-capillary pericytes also dilate (Hall et al., 2014; Kisler et al., 2017; Hartmann et al., 2021b), presumably using different contractile machinery (Hartmann et al., 2021b). This latter study also found these mid-capillary pericytes to show slower constriction to optogenetic stimulation than did αSMA expressing-pericytes.

Vessels also show a transition in expression of elastin across the vascular bed, representing another flow regulation functional transition. In mammals, the number of layers of elastin expressed in the vessel wall scales with blood pressure and arterial size, being highest in the largest arteries (Cocciolone et al., 2018), but with only one layer, just abluminal to endothelial cells, for most intracerebral arterioles (Shinaoka et al., 2013). Elastin expression terminates before arterioles branch successively to become capillaries, presumably reflecting the lower blood pressures to which these smaller vessels are exposed. Elastin’s main role is to increase vessel distensibility, storing energy in the vessel wall during systole, and releasing it during diastole, thus smoothing blood flow across the heart beat and dampening pressure waves, protecting downstream vascular beds from large fluctuations in pressure (Shinaoka et al., 2013). In addition to this biomechanical role, it may also act as a signaling molecule, regulating and attracting smooth muscle and immune cells (Cocciolone et al., 2018).

Elastin can be readily labeled by i.v. injection or incubation of tissue with AlexaFluor 633 hydrazide (Shen et al., 2012), but its role in microvascular regulation remains understudied. Recently, however, it has been used to identify intracortical arterioles in which endothelial caveolae were found to be critical players in neurovascular coupling (Chow et al., 2020), and was found around precapillary sphincters branching off penetrating arterioles that regulate blood flow and pressure into the cortical capillary network (Grubb et al., 2020; Zambach et al., 2021).

**FIGURE 2:**
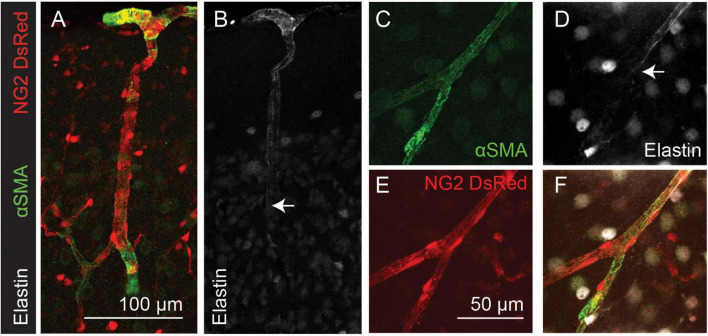
Elastin labeling (white) terminates before αSMA labeling (green) in the cortical microvasculature (arrows). Red: DsRed expressed in NG2-positive mural cells. Panels **(A–F)** show arteriole and capillary example images. Arrows indicate termination point of elastin labeling. Images are representative of 9 (αSMA) and 12 (elastin) vascular trees, which are quantified in Figure 4.

To test whether the transitions between αSMA and elastin expression occur at the same location, thus defining two different vessel “types,” we used immunolabeling for αSMA combined with AlexaFluor 633 hydrazide labeling of elastin. We found that elastin terminated on vessels of large and small diameters (Figures 2B,D) but always upstream from the point of termination of αSMA (Figures 2A-D,F; 4/4 double-labeled branches showed non-overlapping termination points).

### Nestin Expression in the Capillary Bed Extends Beyond αSMA Labeling

**FIGURE 3:**
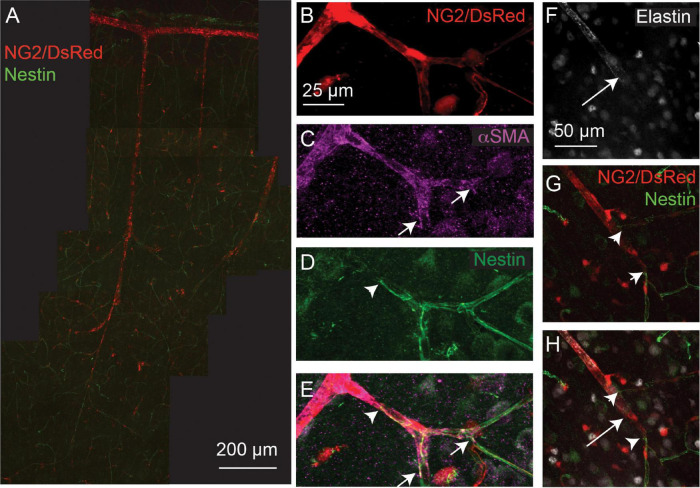
Nestin expression (green) extends from the capillary bed to terminate (arrowheads) on vessels that express αSMA (magenta; termination point showed with small arrows), but does not extend as far as the termination point of elastin (long arrows). Red: DsRed-NG2 positive mural cells. **(A)** Example vascular bed from pia (top) to layer V/VI of cortex. **(B–E)** and **(F–H)** are two other example vessels at higher magnification. Images are representative of 11 vascular trees, which are quantified in Figure 4.

Nestin is an intermediate filament protein, commonly thought to be expressed in proliferating cells (Suzuki et al., 2010), and is associated with cardiovascular remodeling (Calderone, 2018). Recently it has been shown to be expressed in quiescent as well as proliferating endothelial cells and, indeed, may exert an inhibitory effect on endothelial cell proliferation (Dusart et al., 2018). Nestin expression by endothelial cells may therefore not necessarily reflect ongoing angiogenesis, but rather indicate the angiogenic potential of vessels. Double labeling of nestin and αSMA or elastin in NG2-DsRed mice revealed the capillary network to be nestin-positive (Figure 3). Nestin labeling extended from the capillary bed into lower branching order capillaries that were also αSMA positive (Figures 3B-D), but never overlapped with elastin-labeled vessels (Figures 3F-H).

**FIGURE 4:**
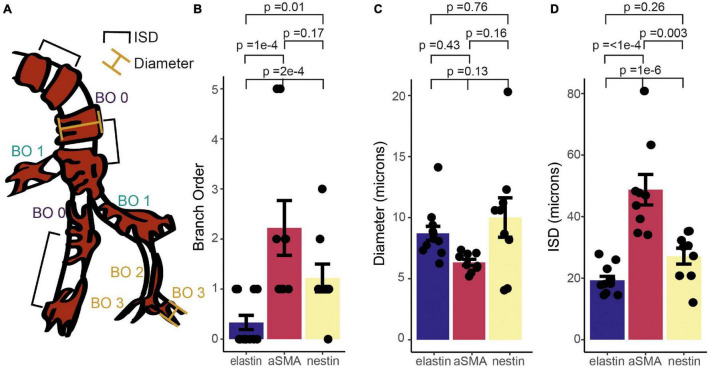
Quantification of relative termination points of three vascular functional markers, assessed from vessel branch order (BO; **A,B**), luminal diameter **(A,C)** and inter-soma distance (ISD; **A,D**). Bars represent mean ± SEM. Each data point represents a vessel (elastin, *n* = 12; αSMA, *n* = 9, nestin, *n* = 11). *P* values are results from independent sample t tests corrected for multiple comparisons using the Holm-Bonferroni method.

To quantify the expression patterns of nestin, αSMA and elastin across the microvascular network, we next categorized the termination points of each label according to vessel lumen diameter, branching order from the penetrating arteriole (where the penetrating arteriole is 0th order; Figure 4A), and the distance between mural cells, or inter-soma distance (ISD). This latter measurement serves as an indicator of the morphology of these cells, as they transition from banded SMCs that are adjacent to each other, to pericytes with distinct soma and processes that become progressively longer along the vascular bed into the capillary network (Grant et al., 2019). Termination points of the different markers occurred at vessels of similar diameters (Figure 4B), but at different branch orders (Figure 4A) or ISD values (Figure 4C), indicative of different positions in the vascular tree. Specifically, elastin termination points were on vessels of significantly lower branch orders than termination points for αSMA or nestin, and mural cells at elastin and nestin termination points were significantly closer together than at termination points for αSMA. Furthermore, consistent with our observations from double labeling of elastin and αSMA (Figure 2), the ISD at which elastin terminated was always smaller than that where αSMA terminated (Figure 4D). Thus, each functional transition occurred at a different point in the vascular tree, as defined by branch order and/or ISD. This means that in addition to the functional transitions between elastin presence and absence, and αSMA presence and absence, there is an additional functional transition point, where nestin terminates, between these two positions.

### nNOS Association With Blood Vessels Is Strongest in Low Branching Order Vessels

**FIGURE 5:**
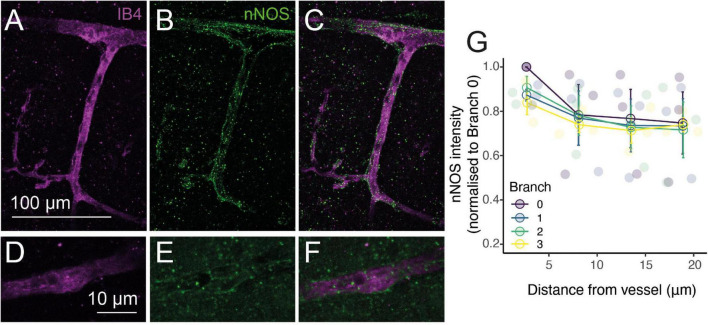
NO production occurs preferentially near vessels. Alexa 647-conjugated Isolectin B4 (IB4; **A,C,D,F**; magenta) and immunohistochemical labeling of nNOS (**B,C,E,F**; green) show increased nNOS expression around vessels. **(G)**: Quantification of nNOS labeling around different vessel branches, expressed by normalizing the intensity of labeling around the vessel to that at the penetrating arteriole (0th order vessel). Data represents mean ± SEM. Individual points show different vessels (*n* = 3).

The vasodilatory signaling molecule nitric oxide (NO) is released by sub-populations of interneurons during synaptic activation, and can mediate or modulate neurovascular coupling (Attwell et al., 2010). In neocortex, if not cerebellum, it seems predominantly involved in regulating arteriole but not capillary diameter, a transition appearing to occur between the diving arteriole and first capillary branch (Hall et al., 2014; Mishra et al., 2016). To investigate whether this reflects neuronal NO sources for vasodilation differing along the vascular tree, we immunohistochemically labeled brain slices for neuronal nitric oxide synthase (nNOS or NOS1) while labeling the vasculature with Alexa647-conjugated isolectin B4, which binds to the basement membrane (Peters and Goldstein, 1979). Consistent with previous studies (Vlasenko et al., 2007), some arterioles showed clear nNOS labeling around (but not within) vessels which extended into the capillary bed, as well as parenchymal signal (Figures 5A-F). To assess whether nNOS is preferentially expressed around particular elements of the vascular network, we measured the intensity of labeling immediately around vessels of different branching orders, and at increasing distances from these vessels (Figure 5G). Linear mixed modeling (with distance from vessel and branch order as fixed factors and vessel as a random factor) showed nNOS labeling to be significantly more intense at the vessel compared to the parenchyma (*F* = 7.20, d.f. = 3,30, *p* = 0.0009), but there was no difference in nNOS labeling around different branches (*F* = 0.87, d.f. = 3, 30, *p* = 0.47), nor did branch order affect the drop-off in signal away from the vessel (*F* = 0.25, d.f. = 9, 30, *p* = 0.98). When just the labeling at the vessel was considered, branch order was borderline-significant (*F* = 4.1, d.f. = 3,6, *p* = 0.068). Together these results suggest that nNOS interneurons do target blood vessels, but that the degree of association is similar between penetrating arterioles and the first few capillary branches from the arteriole. The tendency for marginally stronger labeling at arterioles may contribute to the different dependence of neurovascular coupling reported in capillaries and arterioles (Mishra et al., 2016), but is unlikely to explain it entirely. Interestingly, recent work has shown that NO can modulate the propagation of vasodilation through the vascular network (Kovacs-Oller et al., 2020). It is an open, and important, question if nNOS-derived NO could contribute to neurovascular coupling by modulating the integration of vascular signals across the network in addition to its direct effect on vasodilation, and how such NO release would interact with endothelial (eNOS)-derived NO, which is also an important modulator of neurovascular function (e.g., Toth et al., 2015; Chow et al., 2020). Studying the pattern of targets of NO signaling (e.g., soluble guanylyl cyclase, or cytochrome P450 ω hydroxylase (Sun et al., 2000) should be informative in identifying how different vessels can respond to this perivascular NO production.

### Shifts in Functional Expression Are Not Accompanied by Sudden Shifts in Vascular Cell Length

**FIGURE 6:**
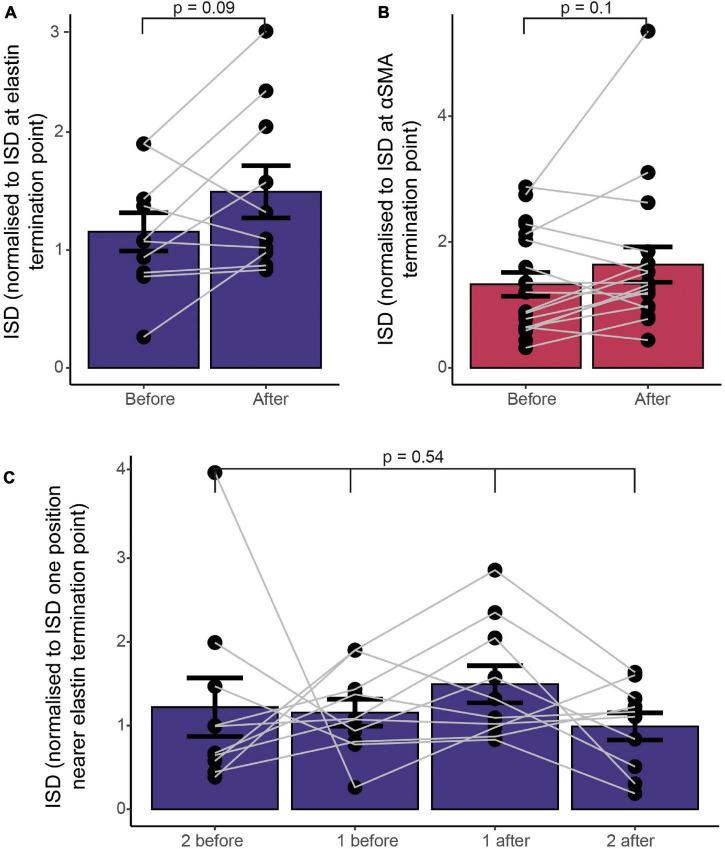
Functional markers terminate at different points along the vascular tree, without a concomitant change in vascular mural cell morphology, as assessed by ISD. **(A)** ISD before or after the termination point normalized to the ISD at the termination point of elastin **(A)** or αSMA **(B)**. Bars show mean ± SEM, with connected data points representing individual vessels (*N* = 9 for αSMA and 11 for elastin). *P* values are from paired *t* tests **(A,B)** or a repeated measures ANOVA **(C)**.

Previous work suggests that mural cell morphology changes in some respects near to the point at which αSMA expression terminates, with αSMA-expressing ensheathing pericytes covering the underlying vessel to a greater degree than mesh pericytes immediately downstream of the αSMA termination point (though the distribution of changes in coverage is shifted to higher branch order vessels than the termination of αSMA; Grant et al., 2019). However, the degree of vessel coverage by processes is not the only morphological change that pericytes undergo across the vascular network, as they change cell length (indicated by ISD), as well as soma orientation and shape, number and orientation of processes and many other descriptors (Zimmermann, 1923). ISD is different across pericyte categories as defined by vessel coverage, with ensheathing pericytes having a lower ISD than mesh pericytes (Grant et al., 2019; Shaw et al., 2021), so we wondered whether any abrupt shifts in ISD would be observed at functional marker transitions, suggestive of a major change in vessel type at this point. We therefore plotted the ISD immediately before and after the termination points of αSMA and elastin, normalized to the ISD at the termination point (Figures 6A,B). There was no significant change in ISD at the termination point of either functional marker. We had measured more ISD values on the elastin vessels, so also calculated the ISD two further cells away from the termination point, which also showed no differences compared to the cells nearer the transition point, further emphasizing the lack of an abrupt change (Figure 6C).

### Mural Cells Are More Densely Spaced at Branch Points Compared With Non-branch Regions

**FIGURE 7:**
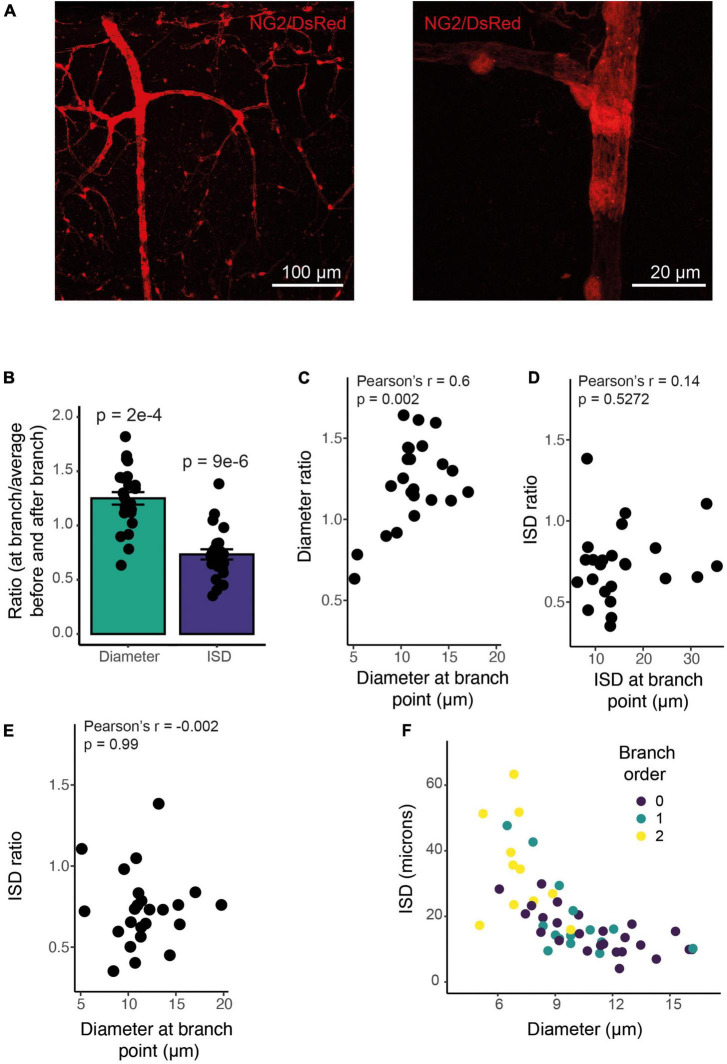
ISD increases along the vessel but is smaller at branch points of any size. **(A)** Example NG2/DsRed labeled cortical vasculature, showing a whole penetrating arteriole and branches (left), and a high-resolution image of a single branch point, with clustered pericytes (right). This whole vascular tree is shown in Supplementary Figure 1. **(B)** Branch points have a larger diameter and more densely spaced pericytes than surrounding non-branch regions: The ratio of diameter at a branch point to the average of values immediately up and downstream of that branch point is larger than 1, and the ratio of ISD at a branch point to the average of ISD up and downstream of that branch point is less than one. *N* = 24 branch points. P values are from one sample t tests compared to 1. **(C)** This diameter ratio correlates significantly with the vessel diameter: smaller vessels show a relatively smaller increase in diameter at branch points. ISD ratio (calculated as in B) is uncorrelated with either ISD **(D)** or diameter **(E)**. Thus, smaller vessels show the same proportional increase in pericyte density at branch points as do large vessels. Bars represent mean ± SEM, data points are individual branches from 16 vessels. **(F)** Mural cell ISD increases with decreasing vessel diameter. Dots represent individual ISD values from 20 vessels.

Pericytes are often found at vessel bifurcations (Hartmann et al., 2015), but we also noticed that they appeared to have a different morphology at branch points, cells being clustered with a shorter ISD and greater vessel coverage (Figure 7A). Indeed, both vessel diameter and ISD were greater at branch points than expected based on the average of the upstream and downstream values of these parameters (Figure 7B). Furthermore, while the increase in diameter was larger at branch points on larger vessels (Figure 7C), the change in morphology, indicated by the relative change in ISD, occurred on all vessels irrespective of the vessel diameter or ISD at the branch point (across a range of vessels from 5-20 μm in diameter; Figures 7D,E). Because ISD generally increases as vessel diameter decreases into the vascular bed (Figure 7F), mural cells at bifurcations therefore, at least in terms of ISD, have a morphology more like larger upstream vessels. This suggests the vascular network shows functional specialization at bifurcations, in addition to classic arteriole-capillary transitions of function. Pericytes at branch points can generate different calcium signals and constriction of different downstream branches, but downstream branch points were less responsive to applied vasoactive agents than upstream vessels (Gonzales et al., 2020). Our data suggest that these downstream bifurcations may still be specialized in some manner, compared to adjacent non-branch capillaries. Pericytes are known to occur frequently at bifurcations (Hartmann et al., 2015). Our analysis reveals that these pericytes at or near branch points are also shorter than expected from the ISD of non-branch pericytes immediately up or downstream.

### *In vivo* Vascular Responses

Functional markers and anatomical changes in mural cell density indicate, therefore, that vascular function changes gradually across the vascular network, with multiple functional transition points corresponding to changes in vascular distensibility, proliferative capacity and contractility. These changes are superimposed on gradually changing mural cell properties that do not show clear alterations (at least in some features) at these transition points, but which do show specializations at branch points that likely reflect functional changes. Furthermore, inspection of the images of DsRed-positive mural cells on arterioles (in Figures 1A, 2A, 3A, 7A and Supplementary Figure 1) show that, unlike commonly assumed, mural cells on arterioles often lose their annular shape as the arteriole dives into the cortex, forming intermediate pericyte morphologies with a distinct soma and processes in deeper regions.

We wanted to test, where possible, how these transitions reflected alterations in the physiological responses of different components of the vascular bed, so studied three properties of visual cortical microvasculature: frequency and size/timing of dilations in response to visual stimulation, and low frequency oscillations in the vasomotion range. We compared responses of the penetrating arteriole before and after smaller branches had come off the main vessel, as well as comparing responses of the arteriole to increasing branch orders of capillaries (see Supplementary Figure 2D for how different vessels’ branch order was defined in order to separate vascular responses along the length of the penetrating arteriole). All data is summarized in Table 1 (and data demographics [cortical depth, diameter] shown in Supplementary Figure 3).

### Arterioles Dilate the Most Near the Cortical Surface but Deeper Sections Behave More Like Capillaries

**FIGURE 8:**
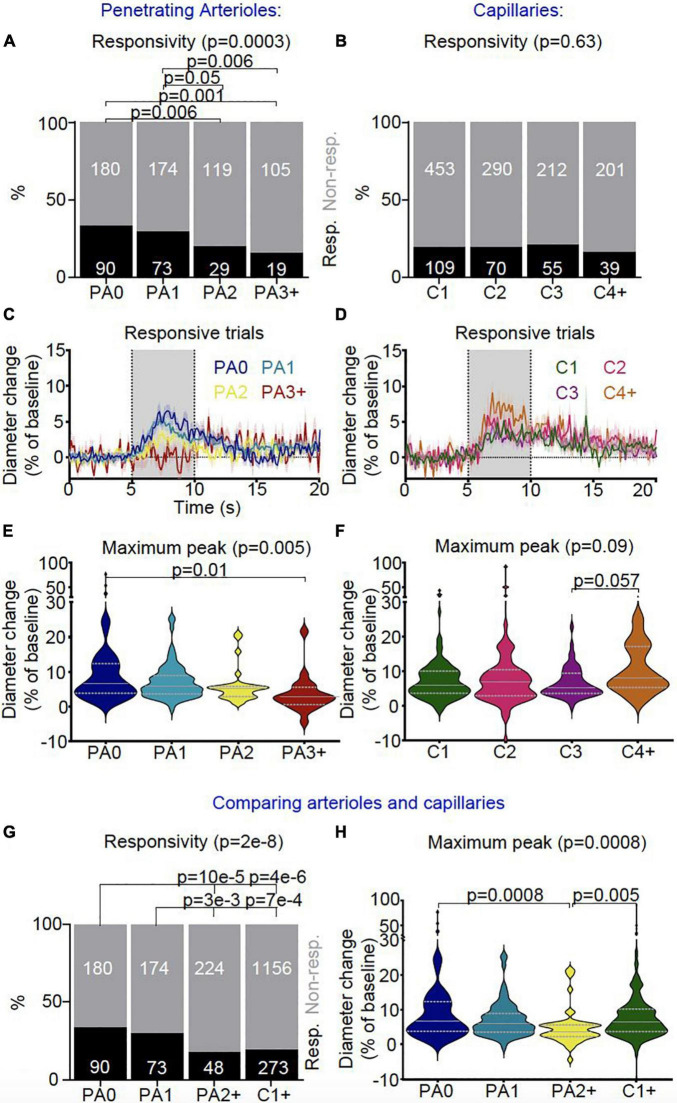
*In vivo* vascular stimulus-dependent responses separated by vessel segment (responsive trials only). Stimulus-induced vascular dilations were classified as responsive (black) or non-responsive across the vascular segments for **(A)** penetrating arterioles (PA0 nTrials = 270, nVessels = 21; PA1 nTrials = 247, nVessels = 22; PA2 nTrials = 148, nVessels = 12; PA3 + nTrials = 124, nVessels = 9) and **(B)** capillaries (C1 nTrials = 562, nVessels = 50; C2 nTrials = 360, nVessels = 29; C3 nTrials = 267, nVessels = 22; C4 + nTrials = 240, nVessels = 21). Lower order (PA0 and PA1) penetrating arterioles were more likely to dilate during stimulus presentation than higher order diving arterioles (PA2, PA3 +), whereas no differences were found in the response rates between capillaries. Vessel responses were plotted for the **(C)** penetrating arteriole (PA0 nTrials = 90, nVessels = 18; PA1 nTrials = 73, nVessels = 15; PA2 nTrials = 29, nVessels = 9; PA3 + nTrials = 19, nVessels = 7) and **(D)** capillary (C1 nTrials = 109, nVessels = 36; C2 nTrials = 70, nVessels = 21; C3 nTrials = 55, nVessels = 17; C4 + nTrials = 39, nVessels = 14) classifications during the stimulus for responsive trials only (traces represent mean ± SEM across trials; data from all trials are shown in Supplementary Figure 4). Gray bar (5-10 s) shows when the stimulus was presented. Responsive trials, averaged across individual vessels, and the maximum dilation during the stimulus presentation was compared between branch orders for **(E)** penetrating arterioles and **(F)** capillaries. There were differences in the maximum dilation during stimulus presentation across sections of penetrating arterioles (*p* = 0.04; Kruskal-Wallis test as data is highly skewed), with the largest dilations in the most superficial section (PA0) (*p* = 0.06 vs PA3 +, pairwise comparison with Wilcoxon Rank Sum test), whereas stimulus-induced dilations were not significantly different across the capillary bed. We then compared vessel responses between the superficial and deep sections of penetrating arterioles (PA0 nTrials = 270, nVessels = 21; PA1 nTrials = 247, nVessels = 22; PA2 + nTrials = 272, nVessels = 21) and the capillary network (C1 + nTrials = 1429, nVessels = 122). **(G)** Lower order (more superficial) penetrating arterioles (PA0-1) were more likely to dilate to visual stimulus than higher order penetrating arterioles (PA2 +) and capillaries (C1 +) (*p* = 2e-8). **(H)** Stimulus-dependent vascular dilation responses were then averaged across each vessel and superficial and deep penetrating arterioles were compared with the capillary bed (PA0 nTrials = 90, nVessels = 18; PA1 nTrials = 73, nVessels = 15; PA2 + nTrials = 48, nVessels = 16; C1 + nTrials = 277, nVessels = 90; data including non-responsive trials is shown in Supplementary Figure 6). The maximum dilation differed across vessel categories (*p* = 0.05; Kruskal-Wallis test), being borderline significantly smaller in deep PA sections than either superficial arteriolar segments or the capillary bed (*p* = 0.06 vs PA0-1 and C1 +). Horizontal gray lines on violin plots show median (solid line) and interquartile range (dotted lines), and statistical comparisons of vessel responsivity rates were made using a Chi-square test with Fisher’s *post hoc* comparison, and of dilation peaks using a Kruskal-Wallis test with *post hoc* Wilcoxon rank sum tests to assess pairwise comparisons.

Vessel responsiveness was assessed across the microvasculature by testing if visual stimulation led to an increase in vessel diameter (of > 0.5 standard deviations of the baseline). Sections of arterioles near the cortical surface dilated significantly more frequently than either downstream arteriole sections (≥ BO2) or downstream capillaries (Figure 8 and Supplementary Figures 4–6). Specifically, descending the penetrating arteriole, dilations occurred with a similar frequency, and responses when they occurred were of a similar size in the first two sections of the vessel (before the first capillary branch and between the 1st and 2nd branches off the penetrating vessel), but these superficial responses were more frequent and tended to be larger than responses of the deepest sections of the penetrating vessel (Figure 8).

Across the capillary bed (i.e., any vessel downstream of the penetrating arteriole, populated by high αSMA ensheathing pericytes or low αSMA mid-capillary pericytes), dilation responses were of a similar frequency and size, with response frequencies being similar to the deep sections of the penetrating vessel (i.e., PA2 + ; Figure 8). There were no differences between the speed of dilations across the different vascular segments (Supplementary Figures 4–6).

### More Arterioles Near the Cortical Surface Show Higher Power in the Vasomotion Range Compared to in Deeper Sections and the Capillary Bed

**FIGURE 9:**
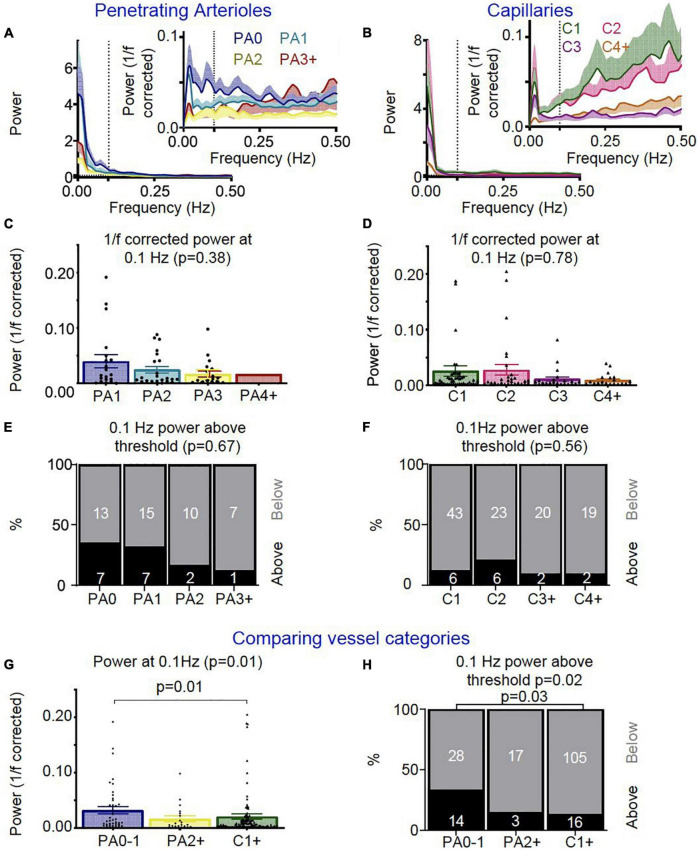
*In vivo* vascular vasomotion responses separated by vessel segment. Average power spectra of **(A)** penetrating arteriole (PA0 nVessels = 20, PA1 nVessels = 22, PA2 nVessels = 12, PA3 + nVessels = 8) and **(B)** capillaries (C1 nVessels = 49, C2 nVessels = 29, C3 nVessels = 22, C4 + nVessels = 21) were separated by branch order for raw traces (left) and 1/f corrected traces (right insert). No significant differences were seen in the 1/f corrected power at 0.1 Hz between **(C)** penetrating arteriole or **(D)** capillary segments. Error bars represent mean ± SEM, and power at 0.1 Hz was compared between individual vessels using a Kruskal Wallis test. **(A)** threshold was set for assessing the number of vessels which showed high 1/f corrected power at 0.1 Hz (threshold: 0.5 * standard deviation across all vessels’ 0.1 Hz 1/f corrected power values), and no significant differences were seen between **(E)** penetrating arteriole or **(F)** capillary vascular segments in the ratio of vessels with higher 1/f corrected power at the vasomotion frequency (numbers in bars represent individual vessels). We then compared vessel responses between the penetrating arterioles (PA0-1 nVessels = 42, PA2 + nVessels = 20) and capillary network (nVessels = 120). As for neurovascular coupling responses, superficial (PA0-1) and deep (PA2 +) arterioles were compared with the capillary bed (C1 +). **(G)** Average 1/f corrected power at 0.1 Hz was different across these vessel groups, with superficial arterioles showing significantly more power at 0.1 Hz than capillaries (*p* = 0.01; Wilcoxon rank sum pairwise comparisons). **(H)** The lowest order penetrating arterioles (PA0-1) also had more vessels with higher 1/f corrected power at 0.1 Hz than other categories (*p* = 0.01), specifically when compared to the capillaries (*p* = 0.03). Statistical comparisons of the number of vessels with higher power in the vasomotion range were made using a Chi-square test with Fisher’s *post hoc* comparison, and of power at 0.1 Hz using a Kruskal Wallis test with Wilcoxon rank sum pairwise *post hoc* tests.

We next measured vasomotion of the vascular diameter of surface and deep sections of penetrating arterioles and different branches of the capillary bed, finding that vasomotion (power at 0.1Hz) was similar between arteriole sections and between capillaries (Figures 9A-F). When we compared arterioles near the cortical surface (PA0-1), to deeper arteriole sections (PA2 +) and capillaries (C1 +), there was some evidence that penetrating arterioles show more vasomotion near the pial surface, as power at 0.1Hz was highest in superficial penetrating arterioles (Figure 9G), and significantly more upstream arteriole sections (PA0-1: 33%) showed an increase in power in the vasomotion range compared to capillaries (C1 + : 13%) (0.5 SD above baseline; Figure 9G,H).

## Discussion

Our results demonstrate the existence of an intermediate transition point in vascular function between the termination points for elastin and αSMA – a transition in nestin expression. Furthermore, pericyte morphology as assessed from ISD does not alter abruptly around these transition points, suggesting that gradual changes in morphology along the vascular network are superimposed upon multiple sharp changes in protein expression levels. In both large and small vessels, branch points are also functionally specialized, having denser pericyte coverage than adjacent up and downstream vessels. Conversely, we did not find any transitions in expression of classic pericyte markers PDGFRβ and NG2, or perivascular nNOS levels, which were expressed from the pia to the mid-capillary bed. Finally, *in vivo*, below their second branch, penetrating arterioles showed similar neurovascular coupling and vasomotion to capillaries, having a lower frequency of dilation or vasomotion compared to the first two segments of the penetrating arterioles.

Thus overall, our data support a view of the vascular network whereby sharp distinctions between different vascular segments do not exist, but rather different functions transition at different positions within the vascular tree, within classic vascular categories such as “arterioles” and “capillaries” as well as between them. This means that vascular function overall changes gradually across the vascular network, including along classically defined single “vessel types” such as diving arterioles.

### Expression of Pericyte Markers Throughout the Vascular Network

We found PDGFRβ and NG2 to be expressed throughout the arteriole-capillary vascular network. Both are classically considered to be pericyte markers (Winkler et al., 2010; Armulik et al., 2011), but our results are consistent with the described effects on smooth muscle cells of PDGFRβ gain-of-function mutations, which increase leukocyte accumulation in the aorta (He et al., 2015). Single cell RNA seq analyses also support the more widespread expression of both PDGFRβ and NG2, with mRNA transcripts found in smooth muscle cells as well as pericytes (Vanlandewijck et al., 2018), albeit at moderately lower levels.

The function of these two “marker” proteins may be different depending on their vascular location. NG2 is known to be important for neovascularization and stabilization of newly formed vessels, *via* the interaction of NG2 with integrins and growth factor receptors on the same and other cells (Stallcup, 2018). In capillary pericytes of the mature vasculature, however, it promotes the formation of new capillaries through angiogenesis, whereas in larger vessels it instead may promote arteriogenetic remodeling of vessel diameter (Rundek and Della-Morte, 2015). Such remodeling can occur after decreased tissue oxygen and, consistent with widespread NG2 expression, cells in surface and penetrating arterioles, and the capillary bed, have been found to proliferate after cerebral ischemia (Wei et al., 2001). PDGFRβ also functions differently in arteries compared to smaller vessels. In culture, pericytes but not smooth muscle cells shed PDGFRβ in response to stress (Sagare et al., 2015) and mutations that block PDGFRβ signaling reduce pericyte number and increase capillary leakiness, without affecting smooth muscle cells (Nikolakopoulou et al., 2017). Conversely, inhibition of PDGFRβ pharmacologically or using siRNA preserves cerebral arterial smooth muscle cells and arterial vascular tone after sub-arachnoid hemorrhage (Shiba et al., 2012; Wan et al., 2019), highlighting a potential pathophysiological contribution of PDGFRβ signaling to arterial smooth muscle cells.

Thus both NG2 and PDGFRβ are expressed throughout the cerebral microvasculature, but differ functionally depending on their location, presumably due to differences in expression levels of other proteins that are localized to different parts of the vascular network.

### nNOS Is Expressed Around Arterioles and Capillaries

Given the involvement of nNOS-derived NO in arteriole but not capillary neurovascular coupling (Mishra et al., 2016), we expected to observe differential expression of nNOS along the vascular network. However, while nNOS was expressed at greater levels around vessels than in the parenchyma, this occurred to a similar degree for all vessels studied (up to 3rd branch order) and not just the diving arterioles. NO can control the electrical coupling of pericytes in the retina (Kovacs-Oller et al., 2020) as well as the production of other vasoactive molecules such as 20-HETE (Liu et al., 2008; Hall et al., 2014), so neuronally derived NO may be released onto all vessels but play a different role at arterioles than capillaries, generating a dilation in the former and modulating the response in the latter. A gradient of expression of the other constitutive NOS isoform, endothelial NOS, has not been reported, and RNA Seq data suggests it is expressed at similar levels in arterial, capillary and venous endothelial cells (Vanlandewijck et al., 2018).

### Correspondence of Elastin and αSMA Labeling to Transitions in Physiological Responses

As previously described, elastin and αSMA labeling in the vascular wall both label arterioles (Shen et al., 2012; Grant et al., 2019) with αSMA labeling extending into the capillary bed (Grant et al., 2019; Chow et al., 2020; Thakore et al., 2021). Here we show these termination points are distinct and non-overlapping, occurring at significantly different branch orders and with different pericyte morphologies as assessed by ISD. The location of elastin labeling quite well matches locations where we see transitions in physiological responses - the second branch off the diving arteriole - below which responses seem more like those in the capillary bed. Of our 12 elastin-labeled vessels, 8 terminated on the penetrating arteriole (75%), of which 5 (42% of the total) terminated before the first branch from the arteriole, i.e., upstream of where we observe a change in response frequencies. Thus, arteriole elastin labeling broadly, but not tightly, corresponds with the superficial part of the diving arteriole where neurovascular dilations and vasomotion were most frequently observed.

Though associated with contractile ability, the termination point of αSMA does not, however, correlate very well with the size or frequency of neurovascular coupling responses or vasomotion, we observed *in vivo*. αSMA universally terminated beyond the penetrating arteriole, but we found no differences in neurovascular response frequency, dilation size or vasomotion between different capillary branching orders (1-4 +), though many fewer of these smaller vessels express αSMA. Furthermore, responses were equally frequent in αSMA-expressing deep sections of the penetrating arteriole as in the capillary bed, and capillary dilations were actually larger than these deep arteriole dilations. This is at odds with previous findings, where dilations in ≥ 4th order vessels were substantially smaller than higher order vessels in whisker barrel cortex of awake mice (Rungta et al., 2021). The reasons for this are unclear. Firstly, we compare both response frequency and response sizes, whereas these two measures are conflated in Rungta et al’s paper. However, as we also saw similar response frequencies and sizes across the capillary bed, this cannot explain why we do not see smaller responses in ≥ 4th order vessels. The cortical area is different (visual vs. somatosensory), and the degree of neuronal stimulation might be different (whole field drifting gratings vs. a single whisker deflection), which could perhaps have an impact on neurovascular coupling. Another potential cause of these differences is the method of detecting vascular diameter. We used xy images from which we calculate the diameter perpendicular to every point of a small length of a vessel’s axis, thus averaging across space (12-109 pixels, or 2.4-22 microns), while Rungta et al. used line scans of vessels to measure vascular diameter at a single position. The spatial smoothing we used in this paper is likely to give us a higher sensitivity to small deflections in diameter.

Previous work in anaesthetized animals, has also found different vascular segments to show different time courses of dilation, with deeper sections of arterioles or first order capillaries responding faster than superficial arteriolar segments (Tian et al., 2010; Hall et al., 2014; Rungta et al., 2018). Our data from awake mice did not show this, with similar response kinetics between vascular segments.

### Transitional Segment?

The vascular segment between the penetrating arteriole, or end of elastin labeling, and the end of αSMA labeling has often been termed a “precapillary arteriole” or a “transitional segment” (e.g., Rungta et al., 2021), representing a region where vascular function transitions between arteriole and capillary. However, our *in vivo* data suggests that this segment, corresponding roughly to branch orders 1 to 3, is not (in our hands) where transitions of contractile behavior occur. Other transitions do occur in this zone: We found endothelial expression of the intermediate filament protein nestin extends out of the capillary bed to a position between the termination points of elastin and αSMA. In the retina, calponin, filamentous microtubules, αSMA, filamentous actin and myosin heavy chain were all also found to change expression levels across this section (Gonzales et al., 2020). However, these all transitioned at different positions, calponin terminating on the arteriole, microtubules on branch 1 and αSMA on branch 2, with filamentous actin and myosin heavy chain gradually decreasing in expression levels from branch orders 0 to 4 (beyond which vessels were not studied). These progressive changes in function across a number of markers fit with the gradual changes in mural cell morphology or ISD we observed before and after the termination points of elastin and αSMA, at roughly branch orders 0 and 3, respectively. Thus this “transitional zone” is not uniform, with a single type of vascular cell and, crucially, is not the only region where such transitions of function are occurring, as similar transitions in vasomotion, neurovascular coupling, and mural cell morphology also occur when descending the penetrating arteriole: Neurovascular coupling responses and vasomotion were observed more frequently in superficial segments of diving arterioles than in either downstream capillaries or deep sections of the arterioles, which were similar to each other in response characteristics. Correspondingly, mural cells lost their annular smooth muscle cell morphology to gain a distinct soma and processes at lower reaches of the diving arterioles. This suggests arteriolar mural cells can be pericytes, unlike has been argued (Hartmann et al., 2021a).

This data suggests that the division of the vascular network into four functional segments: arterioles with SMCs, a transitional zone with ensheathing pericytes, capillaries with capillary pericytes and venules with venular SMCs (Hartmann et al., 2021a), while helpful in discussing broad functional changes across the network, is overly simplistic and neglects the gradual transition in functions that occurs. Indeed, multiple other functional transitions occur at other positions in the vascular tree, including between the pial and penetrating arterioles: Penetrating arterioles exhibit higher contractile tone at low intravascular pressures than pial arterioles (Longden et al., 2016). Neurovascular response sizes are also smaller, possibly because of the lower external pressure on the surface vessel compared to penetrating arterioles which are surrounded by brain tissue (Gao et al., 2015).

### Branch Points May Be Functionally Specialized

Our data suggest that pericytes exist at a higher density at branch points than on surrounding vessel lengths, and this clustering occurs to a similar degree on small and large microvessels. This suggests some functional specialization at branch points. 90% of branch points in the transitional segment were previously found to have a pericyte at that location compared to only 45% of more distal branch points (Gonzales et al., 2020). This corresponds with the increase in pericyte ISD we report here, but our data suggest that even the distal branch points are functionally specialized, as they have a shorter ISD than surrounding non-branch point regions. The pericytes at proximal branch points had calcium sparks that corresponded with selective constriction of individual branches, suggesting branch points serve to direct blood flow to active neurons (Gonzales et al., 2020). Distal branch points (> 4th branch order) were not found to be contractile, and calcium changes in these pericytes were not reported. As our data suggest these distal capillaries do dilate to a similar degree as the proximal branches, it would be valuable to study whether these branch points’ pericyte calcium changes also correspond to changes in vascular diameter of the different downstream branches.

### Transcriptomic Gradients Could Illuminate Transitions in Whole Range of Functions

Our data and the wider literature currently support multiple transitions of function at different positions of the vascular bed, including down diving arterioles, and along increasingly branching capillaries. Contractile function and neurovascular coupling are the functions most widely studied, but transitions at different locations are also seen in oxygen supply as well as expression of transcription factors, transporters, regulators of angiogenesis and immune regulators. However, these studies are all limited by a low capacity to look at different functions. Rather than focusing on individual vascular functions, single cell RNA Seq has the potential to illuminate the whole range of gene expression differences that exist across the vascular network (He et al., 2018). Clustering of vascular endothelial and mural cells has revealed that endothelial gene expression changes gradually, suggesting that there are likely not simultaneous transitions in expression of many genes at the same point on the vessel. More abrupt transitions between smooth muscle cell and pericytes have been reported (Vanlandewijck et al., 2018), which may fit with the sharp transitions observed in some vascular features (e.g., the shift in vessel coverage from ensheathing to mesh pericytes). However, this study also shows large numbers of genes that are expressed in wider zones of the vasculature, e.g., pericytes and arteriolar but not arterial SMCs, supporting the existence of multiple transition points. Indeed, another RNA Seq study identifies multiple pericyte clusters suggestive of multiple functional transition points (Zeisel et al., 2018). Future studies could link single cell RNA seq data to specific spatial locations within the vascular network, to illuminate this issue further. Such data would allow us to better understand the vascular regions to target with pharmacological interventions, given Alzheimer’s disease and stroke are vasculature-degenerating conditions that differentially affect the various parts of the vascular network.

## Conclusion

**FIGURE 10:**
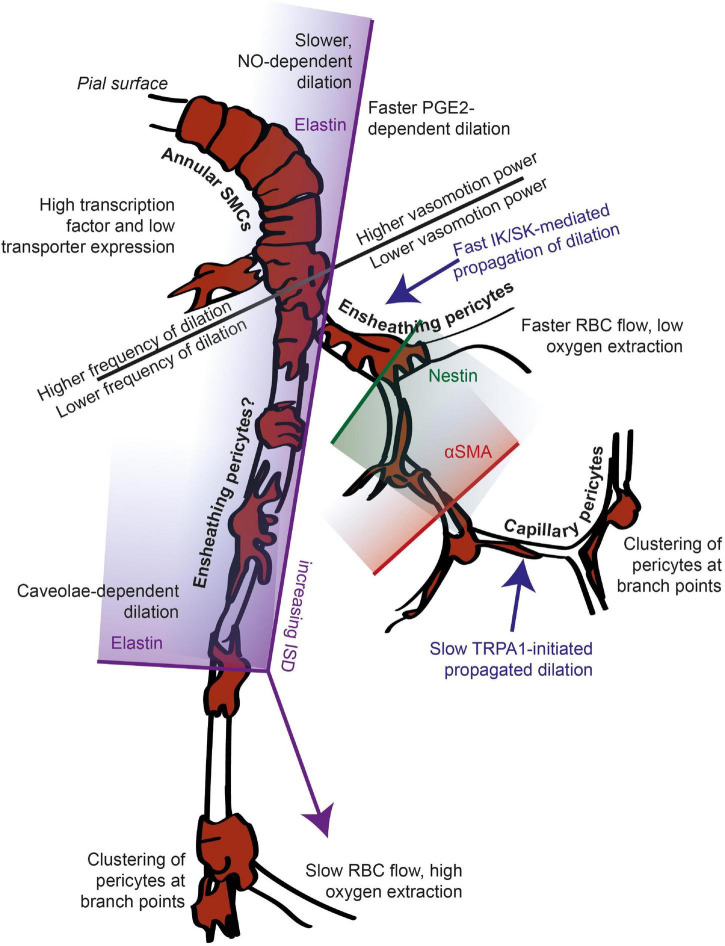
Summary of known functional transition points across the arteriole-capillary axis. The location of many functional transitions remains unknown, indicated by label placement in a general zone, without an arrow (e.g., between slow and fast propagated dilation, and expression levels of transporters vs. transcription factors). Gradient shading shows that the labeled marker is expressed in the vascular zone to the shaded side of the termination point indicated. Current nomenclature for mural cells is indicated, with mural cells on the arteriole suggested to be ensheathing pericytes rather than smooth muscle cells due to their pericyte morphology. This is backed up by the similar behavior of deep arteriole sections compared to the capillary bed, though there are other differences (e.g., elastin labeling, caveloe) as indicated.

Our data, and the literature, support the existence of multiple transition points in vascular function, different proteins being expressed at overlapping sections of the vascular network, and properties of neurovascular and vasomotion response rates, sizes and timings varying at different places in the network (summarized in Figure 10). These various functional transitions are superimposed on gradually changing mural cell morphologies across the vascular tree, which show specializations at branch points. Thus, while categorization of vessels or mural cells may be a useful simplification in some circumstances, it is important to remember that, for example, an upstream ensheathing pericyte is not identical to a downstream ensheathing pericyte, nor to one on a branch point. Understanding where and how different vascular functions (e.g., oxygen and nutrient supply, waste clearance, immune regulation) are supported across the vascular tree is vital to understand how different sections are impacted, and could be targeted, during disease, but will likely require approaches such as spatially-localized RNA Seq to identify how the transcriptome as a whole alters across the cerebral microvascular network.

## Data Availability Statement

The datasets presented in this study can be found at Figshare.com, doi: 10.25377/sussex.17840939.

## Ethics Statement

All experiments were carried out in compliance with the UK Animal Experiments (Scientific Procedures) Act 1986 after approval of the local University of Sussex or UCL Local Ethics Committees and under project or personal licences granted by the UK Home Office.

## Author Contributions

KB, MH-H, DA, and CH collected the data. KS, KB, MH-H, DA, and CH analyzed the data. KS, SA, OB, and CH wrote the manuscript. All authors contributed to the article and approved the submitted version.

## Conflict of Interest

The authors declare that the research was conducted in the absence of any commercial or financial relationships that could be construed as a potential conflict of interest.

## Publisher’s Note

All claims expressed in this article are solely those of the authors and do not necessarily represent those of their affiliated organizations, or those of the publisher, the editors and the reviewers. Any product that may be evaluated in this article, or claim that may be made by its manufacturer, is not guaranteed or endorsed by the publisher.
