# Supplementary material for: Gradual Not Sudden Change: Multiple Sites of Functional Transition Across the Microvascular Bed
Source: Front Aging Neurosci. 2022 Feb 14;13:779823. doi: 10.3389/fnagi.2021.779823 (PMC8885127; doi:10.3389/fnagi.2021.779823)
Supplement: Supplementary file 1 [file Data_Sheet_1.pdf]

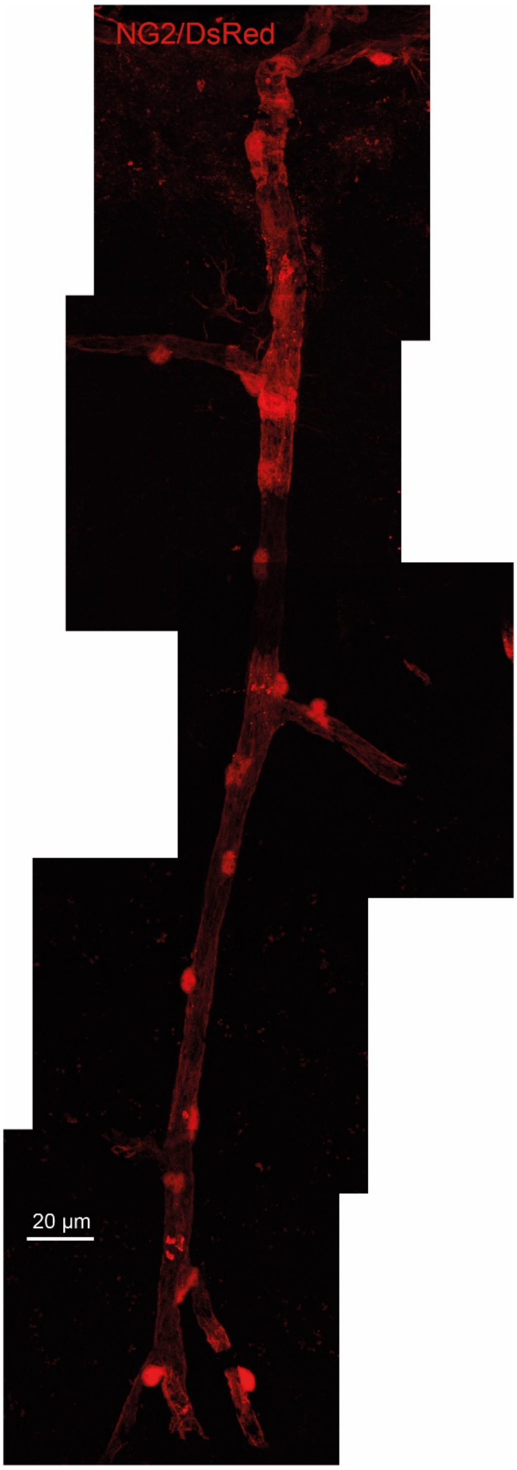

Figure S1. Ex vivo projection of an NG2/DsRed labelled diving arteriole with capillary offshoots. For penetrating arterioles, branch order started at 0 at the pial surface, and increased by 1 down the length of the diving vessel after a branch offshoot was encountered. For capillaries, the first offshoot protruding off the penetrating arteriole was always given a branch order of 1 and vessel branch order increased by 1 for each bifurcation encountered. Scale bar represents 20 µm.

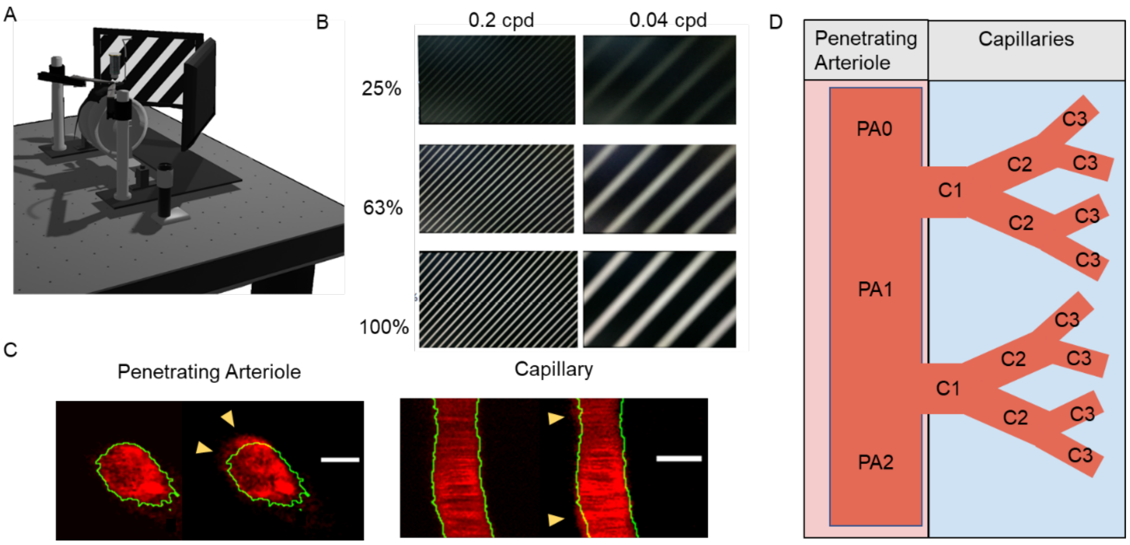

Figure S2. In vivo experimental set-up and branch order classification system. (A) Schematic of awake in vivo imaging set-up, with screens delivering visual stimuli. **(B)** Full screen examples of the contrast (%) and spatial frequency (cpd) variations presented for the visual stimuli. **(C)** Representative two-photon images of a penetrating arteriole (left, scale bar is 10 µm) and capillary (right, scale bar is 5 µm) before and during a vessel dilation (vessel outline from before dilation (left vessel for both vessel types) is shown in green). Vessel dilations are indicated by yellow arrowheads. **(D)** Schematic demonstrating the classification method for branch order labelling in the penetrating arteriole (left, red background) and capillary bed (right, blue background) in vivo data. For the penetrating arteriole, branch order started at 0 and increased by 1 after each branch offshoot. For the capillaries, the first branch immediately attached to the penetrating arteriole was classed as branch order 1, and then branch order increased by 1 after each branch bifurcation.

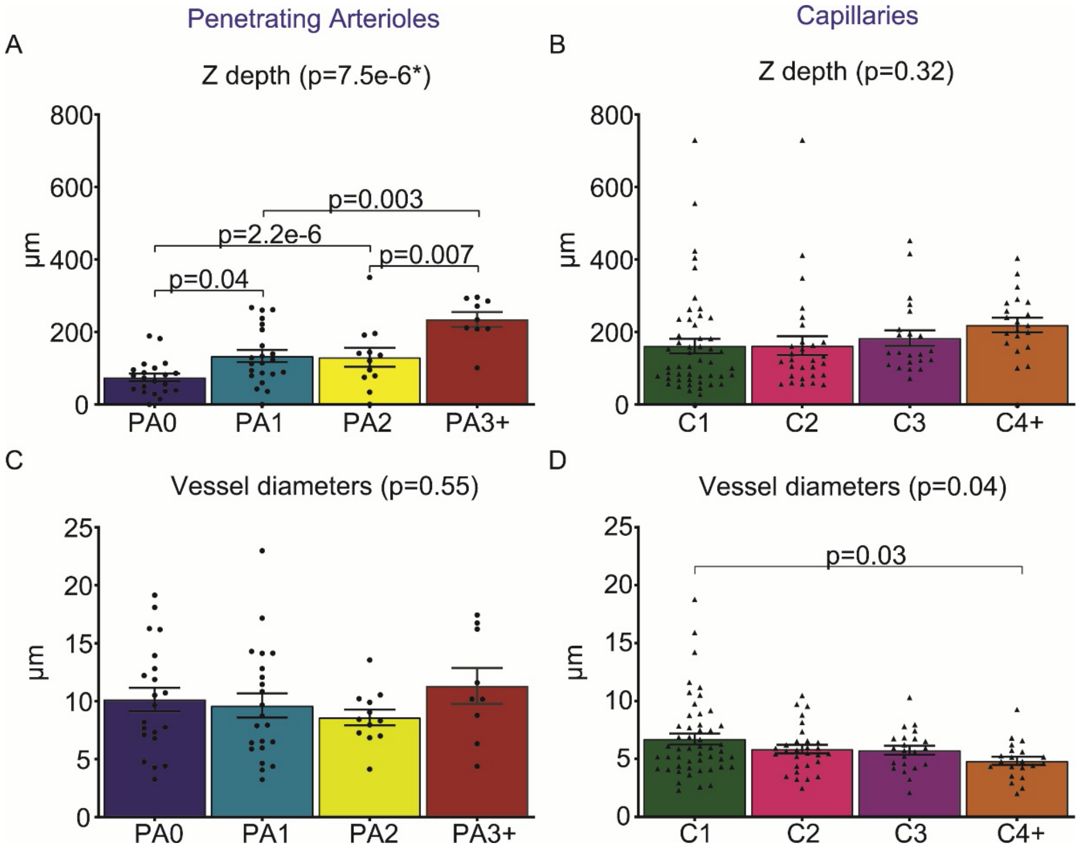

Figure S3. Z depth and vessel diameter ranges separated by branch order. Top row: single dots represent the depth from the pial surface for each individual (A) penetrating arteriole or (B) capillary, as separated by branch order classification (vessel numbers are specified in Table 1 and Figure 8). For the penetrating arterioles there is a pattern of increasing depth from the surface as the branch order increases (p=7.5e-6), which does not hold for capillaries (p=0.32). Bottom row: single data points represent vessel diameters for all individual (C) penetrating arterioles and (D) capillaries recorded per vascular segment. The size of the penetrating arteriole does not change as the depth/branch order increases, whereas first order capillaries are generally larger than the lowest order capillaries (p=0.03). Bar charts represent mean +/- SEM, and statistical tests were an ANOVA with Tukey's post-hoc pairwise test for comparisons between individual branch orders.

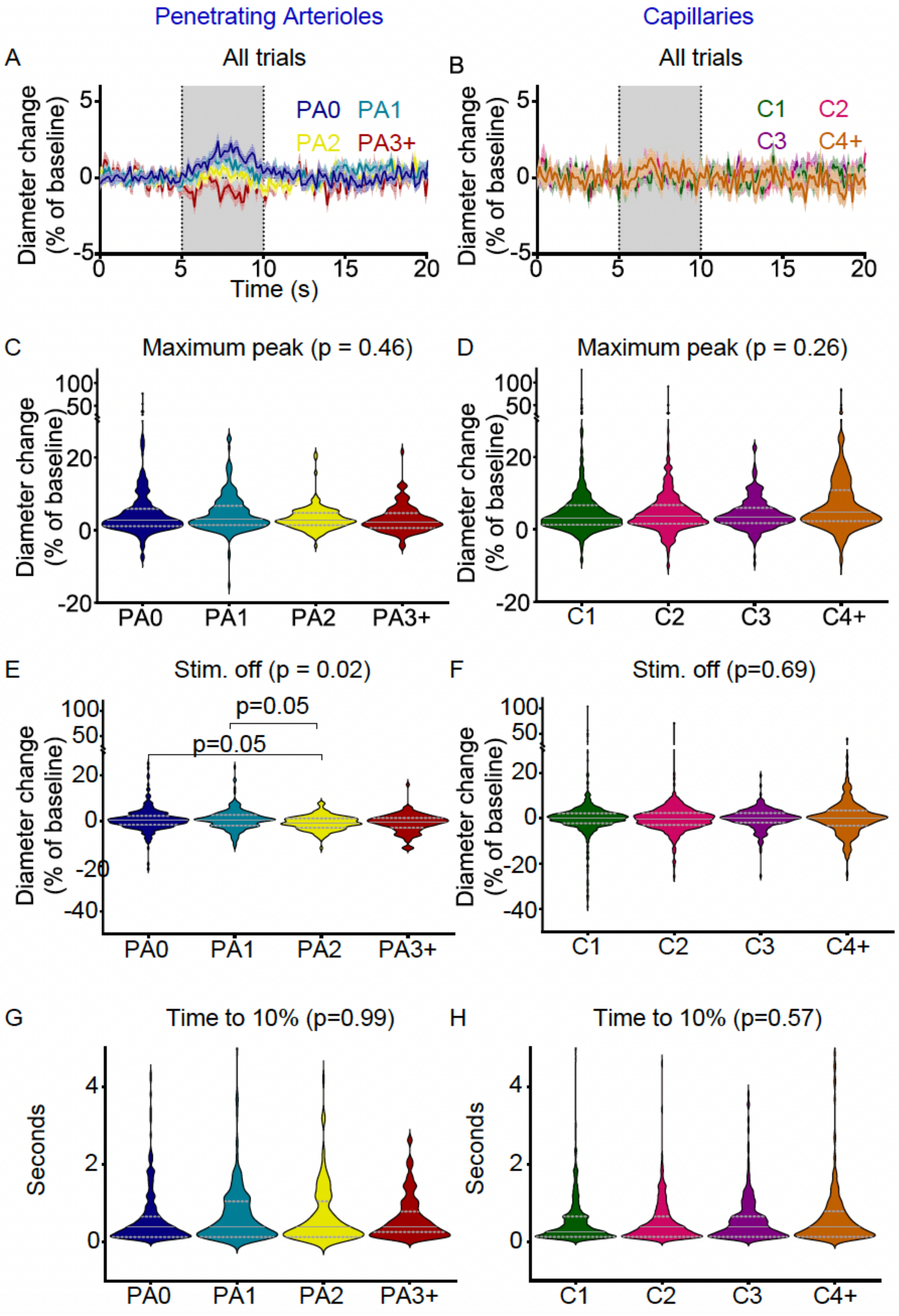

Penetrating Arterioles
Capillaries
A
All trials
B
All trials
PA0 PA1
PA2 PA3+
C1 C2
C3 C4+
Diameter change (% of baseline)
Time (s)
C
Maximum peak (p = 0.46)
D
Maximum peak (p = 0.26)
PA0 PA1 PA2 PA3+
C1 C2 C3 C4+
E
Stim. off (p = 0.02)
p=0.05
p=0.05
F
Stim. off (p=0.69)
G
Time to 10% (p=0.99)
H
Time to 10% (p=0.57)
Seconds

Figure S4. In vivo vascular stimulus-dependent responses separated by vessel segment (for all trials). Vessel responses were plotted across all recorded trials for (A) penetrating arterioles (PA0 nTrials=270, nVessels=21; PA1 nTrials=247, nVessels=22; PA2 nTrials=148, nVessels=12; PA3+ nTrials=124, nVessels=9) and (B) capillaries (C1 nTrials=562, nVessels=50; C2 nTrials=360, nVessels=29; C3 nTrials=267, nVessels=22; C4+ nTrials=240, nVessels=21) during the stimulus (traces represent mean +/- SEM across trials). Grey bar (5-10 seconds) shows when the stimulus was presented. All stimulus-dependent vascular dilations were compared on individual trials between branch orders for each vessel type (left: penetrating arteriole, right: capillary) on the maximum dilation reached during the stimulus presentation (C-D), the value of the dilation response at the time the stimulus ended (10 seconds, E-F), and the time taken (seconds) to reach 10% of the maximum peak value (time to onset, G-H). When considering all trials and not just responsive trials, no differences between arteriole or capillary segments were observed. Lower order penetrating arterioles (PA0-1) showed more sustained dilations than at the higher order (PA2), as dilations were larger at 10 seconds when the stimulus presentation ended, $p=0.02$). Horizontal grey lines on violin plots show median (solid line) and interquartile range (dotted lines), and statistical comparisons were made using a Kruskal Wallis test with Wilcoxon rank sum paired post hoc comparisons.

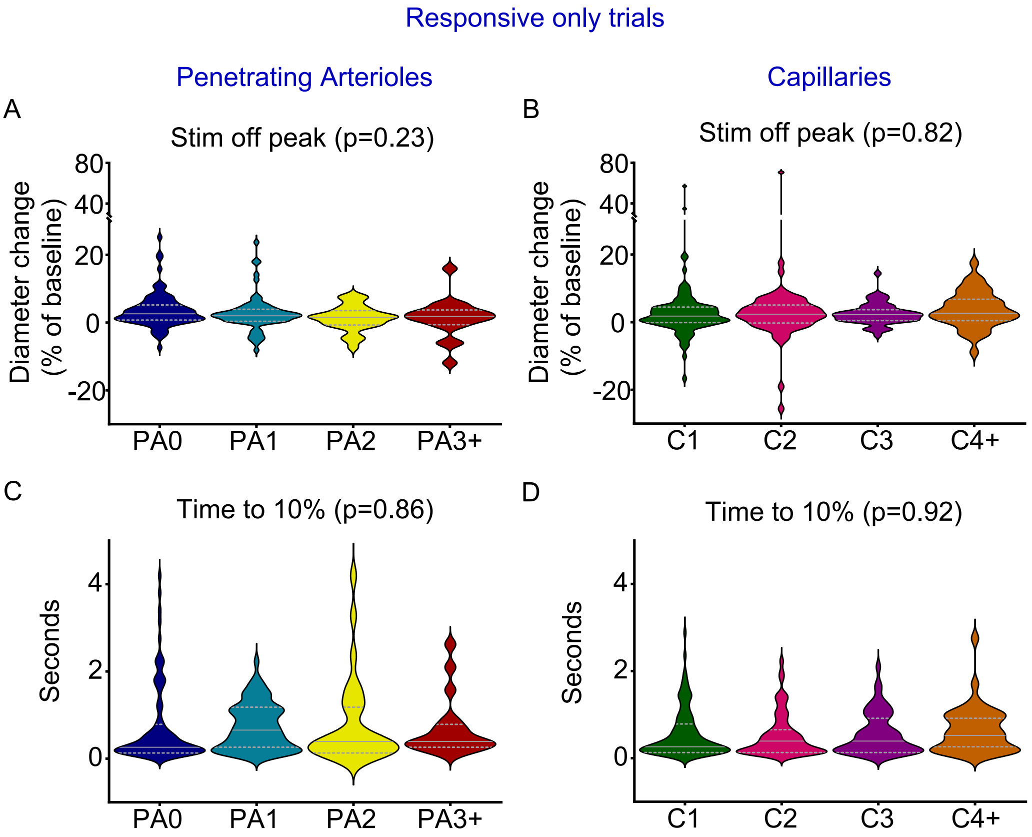

Figure S5. Additional time series comparisons for in vivo vascular stimulus-dependent responses separated by vessel segment (for responsive trials only). When we compared the value of the dilation response at the time the stimulus ended (10 seconds) in responsive trials only for the (A) penetrating arterioles and (B) across the capillary network no significant differences were observed in the penetrating arterioles; number of individual trials and vessels included are specified in Figure 8 legend). When we compared the onset time (time to 10% of the maximum peak during stimulation) for vessel dilations in responsive trials only for the (C) penetrating arterioles and (D) across the capillary network no significant differences were observed Horizontal grey lines on violin plots show median (solid line) and interquartile range (dotted lines), and statistical comparisons were made using Kruskal Wallis tests.

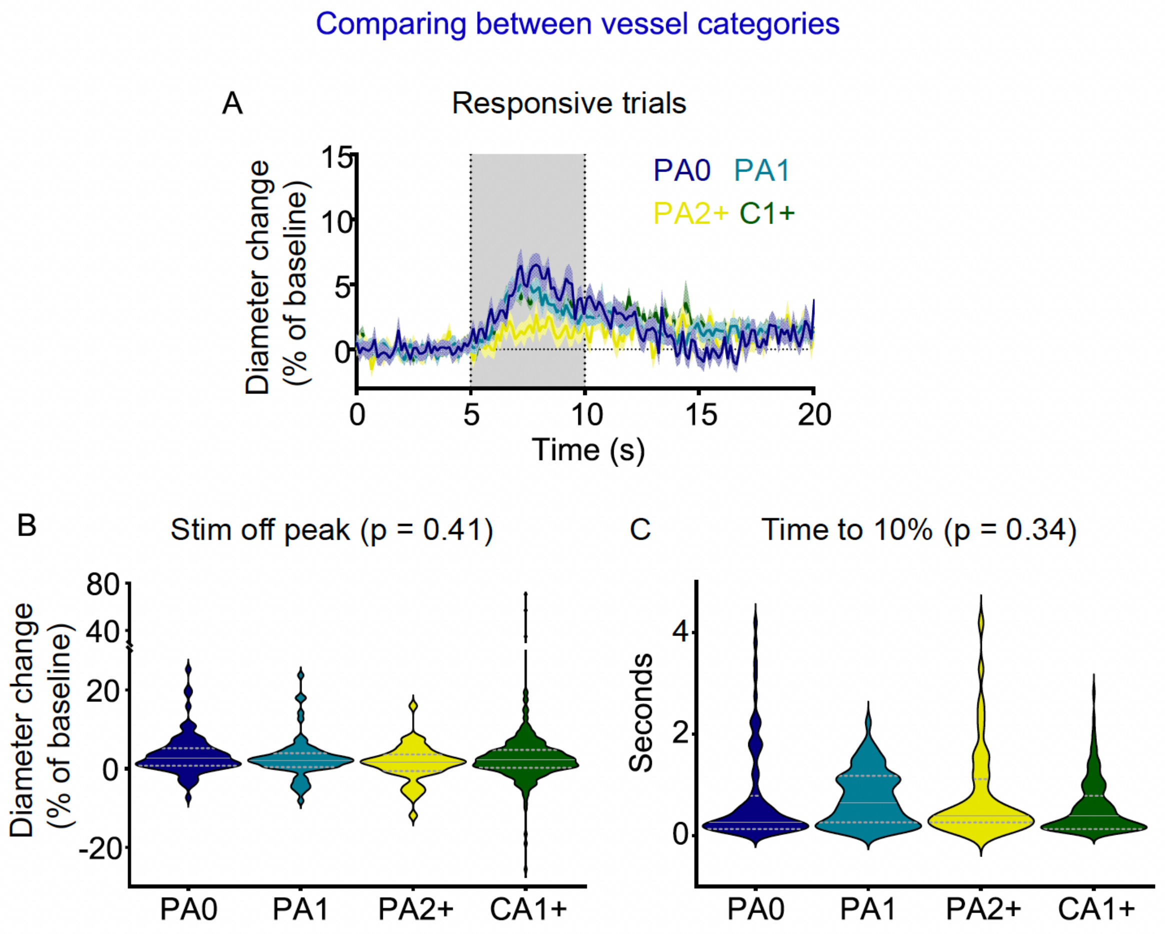

Figure S6. Comparing stimulus-dependent vascular responses between penetrating arterioles and capillaries for responsive trials only. (A) Stimulus-dependent vascular dilations are shown for responsive trials only for the penetrating arteriole (PA0, PA1, PA2+) and capillary (C1+) groups (traces represent mean +/- SEM across trials; PA0 nTrials=90, nVess=18; PA1 nTrials=73, nVessels=15; PA2+ nTrials=48, nVessels=16; C1+ nTrials=273, nVess=88). Grey bar (5-10 seconds) shows when the stimulus was presented. (B) There were no significant differences in the size of the dilation at the stimulus-off point or (C) in the onset time of vessel dilations (time to 10% of max peak). Horizontal grey lines on violin plots show median (solid line) and interquartile range (dotted lines), and statistical comparisons were made on individual trials using a Kruskal Wallis test.
